# Crystal Facets‐Activity Correlation for Oxygen Evolution Reaction in Compositional Complex Alloys

**DOI:** 10.1002/advs.202404095

**Published:** 2024-07-23

**Authors:** Hui‐Feng Zhao, Jun‐Qing Yao, Ya‐Song Wang, Niu Gao, Tao Zhang, Li li, Yuyao Liu, Zheng‐Jie Chen, Jing Peng, Xin‐Wang Liu, Hai‐Bin Yu

**Affiliations:** ^1^ Wuhan National High Magnetic Field Center & School of Physic Huazhong University of Science and Technology Wuhan 430074 China; ^2^ State Key Laboratory of Materials Processing and Die and Mould Technology School of Materials Science and Engineering Huazhong University of Science and Technology Wuhan 430074 China; ^3^ Shenzhen Key Laboratory of Energy Materials for Carbon Neutrality Shenzhen Institutes of Advanced Technology Chinese Academy of Sciences Shenzhen 518055 China

**Keywords:** density functional theory, electrocatalysis, medium‐entropy alloys, oxygen evolution reaction, single crystal

## Abstract

Compositional complex alloys, including high and medium‐entropy alloys (HEAs/MEAs) have displayed significant potential as efficient electrocatalysts for the oxygen evolution reaction (OER), but their structure‐activity relationship remains unclear. In particular, the basic question of which crystal facets are more active, especially considering the surface reconstructions, has yet to be answered. This study demonstrates that the lowest index {100} facets of FeCoNiCr MEAs exhibit the highest activity. The underlying mechanism associated with the {100} facet's low in‐plane density, making it easier to surface reconstruction and form amorphous structures containing the true active species is uncovered. These results are validated by experiments on single crystals and polycrystal MEAs, as well as DFT calculations. The discoveries contribute to a fundamental comprehension of MEAs in electrocatalysis and offer physics‐based strategies for developing electrocatalysts.

## Graphical abstract

Chemically complex materials are attractive for electro‐catalysis for OER, yet their active crystal facets remain elusive. This study demonstrates that the {100} facet of a FeCoNiCr exhibits the highest activity due to low in‐plane density and surface reconstruction, leading to amorphous structures with true active species.

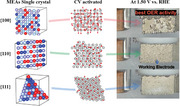

## 1 Introduction

The utilization of renewable electricity for the production of fuels using abundant resources has garnered significant attention in addressing concerns regarding the sustainability of energy and materials.^[^ ^1^, ^2^, ^3^ ^]^ The oxygen evolution reaction (OER) is one of the cornerstones of renewable energy technology but requires efficient catalysts to accelerate its sluggish kinetics.^[^ ^4^, ^5^, ^6^ ^]^ Extensive efforts have been dedicated to the search for suitable electrode materials that can enhance OER performance.^[^ ^7^, ^8^, ^9^ ^]^

Compositional complex alloys, such as high and medium‐entropy alloys (HEAs and MEAs) represent a novel category of metallic materials characterized by multiple principal elements in equilateral or near‐equilateral ratios.^[^ ^10^, ^11^ ^]^ In recent years, there has been a growing interest in developing HEA and MEA electrocatalysts for OER.^[^ ^12^, ^13^, ^14^ ^]^ Various HEA and MEA‐based materials have been investigated and have demonstrated exceptional activity and stability toward OER.^[^ ^15^, ^16^, ^17^ ^]^ However, the underlying mechanism contributing to their exceptional performance remains unclear, and the relationship between their structure and activity has yet to be fully established. Notably, HEAs and MEAs typically serve as pre‐catalysis for OER, as activated samples have shown surface restructuring and even amorphization.^[^ ^18^, ^19^, ^20^ ^]^

One fundamental question in heterogeneous catalysis is determining which crystal facets exhibit the highest level of activity. This question is important for understanding the mechanism and designing materials. Crystal‐facets engineering, in addition to composition manipulation, plays a central role in modulating the kinetics of redox reactions on catalyst surfaces.^[^ ^21^, ^22^, ^23^, ^24^, ^25^, ^26^, ^27^ ^]^

Typically, high‐index facets, denoted by Miller indices {hkl} with at least one index larger than unity, can provide more favorable surface atomic structures, including an increased number of atomic steps, compared to low‐index facets.^[^ ^28^, ^29^ ^]^ However, high‐index facets are unstable due to their high surface energy and tend to rapidly evolve and disappear during material synthesis or catalytic action. Despite this, the impact of crystal facets on OER has not been fully clarified. For example, in the case of Co_3_O_4_, Liu et al observed that the {111} facets exhibited the highest OER activity.^[^ ^30^ ^]^ This was attributed to the presence of a high dangling bond density and surface energy on the {111} facets, which promotes the adsorption and activation of oxygen species involved in the OER. On the other hand, in Fe_2_O_3_, Wu et al found that the (012) facets displayed favorable OER activity.^[^ ^31^ ^]^ This was explained by the decreased occupancy of the surface Fe anti‐bonding orbitals, which enhances the reactivity of the surface toward oxygen species. In the case of MnO, the (100) facets were suggested to have higher OER activity due to the higher adsorption energy of oxygen species on these facets.^[^ ^32^ ^]^ The stronger adsorption of oxygen species facilitates their activation and subsequent OER. Table S1 (Supporting Information) summarizes more examples of how the crystal facets influence OER. It is important to note that these findings are specific to the materials studied and cannot be directly applied to other materials. The OER activity of different facets can be influenced by various factors, including surface energy, atomic structure, electronic properties, surface moieties, and the specific reaction mechanism involved.^[^ ^33^, ^34^, ^35^, ^36^ ^]^

For HEAs and MEAs, there are rare studies on the effects of crystal facets on OER, possibly due to the challenges in preparing samples of single crystals with well‐defined facets. In this work, we fabricated bulk single‐crystal MEAs and investigated the correlations between facets and activity. Our findings demonstrate that, for the FeCoNiCr MEA with face center cubic (FCC) structure, the lowest index facets {100} exhibit the highest activity for OER. We reveal that the underlying mechanism is attributed to the ease of surface reconstruction and formation of amorphous structures.

## 2 Results

### 2.1 Facets‐Activity Correlation in Single Crystal MEA

**Figure 1:**
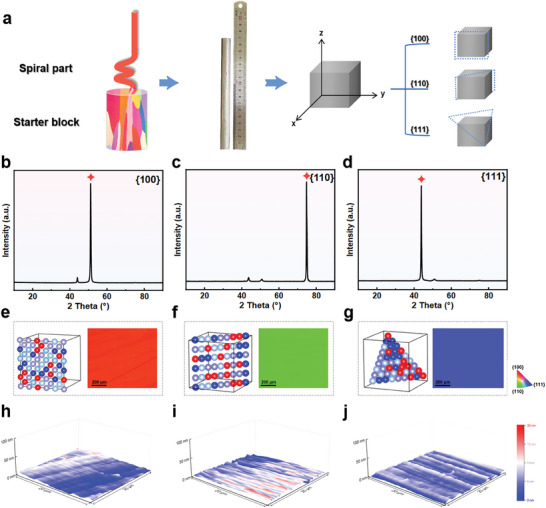
The acquisition and orientation of the single crystal. a) The manufacture of three typical single crystals. b–d) XRD patterns of{100}, {110}, {111}. e–g) Electron Backscatter Diffraction micro‐structures of {100},{110}, and {111}, h–j) Pristine surface morphology of of {100},{110}, and {111} observed by AFM.

Considering the advantages of high catalytic activity, cost‐effectiveness, and environmental sustainability, FeCoNiCr as a model system becomes ideal for scalable and efficient applications.^[^ ^14^, ^15^, ^20^, ^37^ ^]^ Several HEAs (such as FeCoNiCrMn, FeCoNiCrAl*_x,_* and FeCoNiCrCu) contain this composition. We prepared single crystals of FeCoNiCr using the zone melting method, as illustrated in **Figure** **1**a and described in the Methods section. Different facets were prepared by cutting from the single crystal using electrical discharge machining.

Since this single crystal possesses FCC structure, only three important families of facets exist, namely the {100}, {110}, and {111} facets. Figure 1b–d displays the X‐ray diffraction (XRD) patterns for different crystallographic orientations of FeCoNiCr. All XRD patterns exhibit the same FCC structure. The dominant peak and distinct single‐crystal diffraction line in each sample's diffraction pattern confirm the desired orientation and single‐crystal structure.

Electron backscatter diffraction (EBSD) was employed to reconfirm the crystal facets. Figure 1e–g presents the corresponding inverse pole figure (IPF) maps for each crystal facet. We find that each crystal face exhibits a unique and uniform color, which reflects the consistency of the orientation within the crystal. Through these color differences, different crystal faces can be clearly distinguished. For example, a red region may represent a crystal face with a crystallographic orientation of {100} facets, while green and blue regions correspond to two different crystallographic orientations of {110} and {111} facets, respectively. Additional structural characterizations are given in Figure S1–S4 (Supporting Information). Moreover, the original surface morphology of each crystal plane has been confirmed via atomic force microscopy (AFM), and it can be observed that the single crystal surface is smooth (Figure 1h–j). Consequently, we successfully obtained single crystals with well‐defined facets.

**Figure 2:**
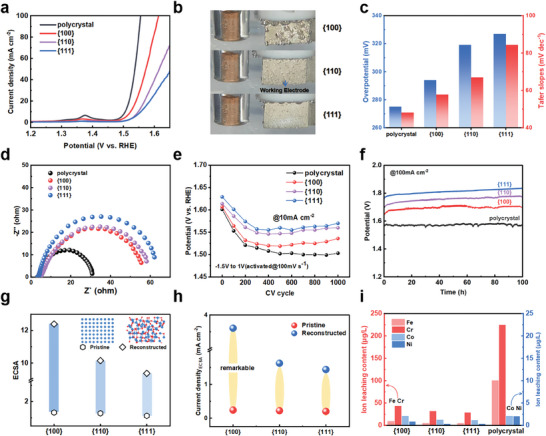
OER performance. a) OER polarization curves. b) OER performance of different crystal facets at 1.5 V versus RHE. c) Overpotential and tafel slope. d) Electrochemical impedance spectroscopy. e)The CV activation process of 100 mV s^−1^. f) Catalytic stability measured by chronopotentiometry in 1 m KOH at room temperature. g)The value of ECSA. h)The experimentally measured normalized current density (@ 1.50 V versus RHE) of the pristine and reconstructed. i) ICP‐MS analysis of electrolyte solutions after reconstruction.

To evaluate OER activity, electrochemical examinations were performed on single crystal FeCoNiCr facets ({100}, {110}, and {111} facets) and a polycrystal. **Figure** **2**a shows the linear sweep voltammetry (LSV) polarization curves that assessed the OER activity of each crystal facet, indicating the correlation between facets and activity. Figure 2b displays the photograph for OER on the catalyst surface at 1.5 V versus RHE, clearly showing that {100} facets exhibited the most pronounced and vigorous oxygen bubbles.

Figure 2c illustrates the key indicators used to evaluate electrocatalytic activity, including overpotential and Tafel slope. Impressively, the {100} facet shows greatly enhanced electrocatalytic activity compared to {110} and {111} facets, with an overpotential of 294 mV required to reach the current density of 10 mA cm^−2^. Tafel slope was employed to determine the electron transfer kinetics during OER, with the {100} facets exhibiting a smaller Tafel slope of 57.8 mV dec^−1^, indicating faster kinetics for OER.

To further investigate the electrochemical reaction kinetics during the OER process, we conducted electrochemical impedance spectroscopy (EIS) (Figure 2d). The Nyquist plot reveals that {100} facet exhibits the lowest charge transfer resistance, significantly lower than {110} and {111} facets. This indicates that the {100} facet possesses the highest conductivity and fastest charge transfer, likely attributed to an increased density of active sites. These findings collectively suggest the distinct OER activities of the {100}, {110}, and {111} facets, with the {100} facets exhibiting superior catalytic activity compared to {110} and {111} facets.

In practical applications, the long‐term durability of electrocatalysts is crucial.^[^ ^4^ ^]^ The long‐term cyclic voltammetry (CV) activation process can simulate the continuous stresses and environmental influences experienced by the catalyst in real‐world applications, providing a more comprehensive understanding of stability performance. The CV activation process was conducted using a potential scan rate of 100 mV s^−1^ in 1 m KOH electrolyte at room temperature, with a potential window of −1.5 to 1 V_Hg/HgO_. As shown in Figure 2e, the OER activity of {100}, {110}, and {111} facets gradually increases over the CV cycles and stabilizes after ≈300 cycles. For the single crystal, the {100} facet exhibits the best OER performance after 300 cycles. From cycles 300 to 1000, the activity of each facet remains almost unchanged, indicating the good durability of the single crystal catalysts. To further investigate the stability of polycrystal, {100}, {110}, and {111} facets, we conducted chronoamperometry experiments. As shown in Figure 2f, at a current density of 100 mA cm^−2^, the potential changes slightly after 100 h of testing. This indicates excellent long‐term stability of both polycrystal and the three single‐crystal catalysts.

The above results illustrate a correlation between crystal facets and OER activity. Typically, crystal facets with more active species exhibit higher catalytic activity. Firstly, measurements of the electrochemical active surface area (ECSA) indicate that the ECSA value of {100} is greater than {110} and {111} facets both before and after CV activation, with the most significant enhancement observed for {100} facets (see Figure 2g; Figures S7 and S8, Supporting Information). This suggests that {100} facets generate more active species compared to {110} and {111} facets. Further analysis was conducted on the intrinsic activity of different crystal facets, evaluating it based on ECSA. The {100} facet showed lower overpotential and higher C_dl_ compared to the {110} and {111} facets (Figure S6, Supporting Information). Moreover, measurements of pristine and CV‐activated normalized current densities (@ 1.50 V vs RHE), show remarkable changes on {100} facets (Figure 2h).

In addition, we find that the effective formation of active species on the electrode surface is related to the leaching of metal elements.^[^ ^38^ ^]^ This view is supported by inductively coupled plasma mass spectrometry (ICP‐MS) of the electrolyte used after the activation process (Figure 2i). During the CV activation, the leaching of Fe and Cr leads to the gradual exposure of Ni and Co species which have higher resistance to leaching. The leached Fe gradually accumulates in the form of active Fe within the active oxides of Ni and Co.^[^ ^39^, ^40^ ^]^ Cr precipitates in the form of CrO_4_^2−^ dissolved in the electrolyte, the leaching of Cr enhances the trend of surface reconstruction, thereby activating it into highly active metal oxides.^[^ ^41^ ^]^

By comparing the leaching of elements on different crystal faces, we study the single crystal's ability to reconstruct and the number of active substances generated after CV activation.^[^ ^38^, ^42^, ^43^ ^]^ It can be observed that Fe and Cr in the polycrystal exhibit the most significant leaching, primarily due to grain boundaries.^[^ ^14^ ^]^ In the single crystals, {100} facet exhibits the highest leaching of Fe and Cr, followed by {110} facets, while {111} facets shows the least leaching of elements. Additionally, AFM (Figure S10, Supporting Information) of the surfaces after electrochemical activation demonstrates that the {100} facet becomes rougher compared to the {110} and {111} facets after activation. This indicates that the {100} facet undergoes more pronounced surface reconstruction under the same electrochemical activation conditions. Overall, the different structures of crystal facets result in varying restructuring abilities, with low in‐plane density {100} facets being easier to restructure during the activation process.

### 2.2 Validation with Polycrystals

We specifically chose polycrystal samples as experimental materials. The use of polycrystals is important as they contain a continuous range of facets and orientations. This property not only validates the phenomena observed in single crystal samples but also provides a unique opportunity to explore and identify other crystal facets that may exhibit higher OER activity. Additionally, we can assess the impact of grain boundaries on the overall results. We observed that the overpotential and Tafel slopes in the polycrystal samples were smaller than any of the studied single‐crystal facets, as shown in Figure 2c. This indicates that the grain boundaries contribute significantly to the observed OER activity.^[^ ^14^ ^]^

**Figure 3:**
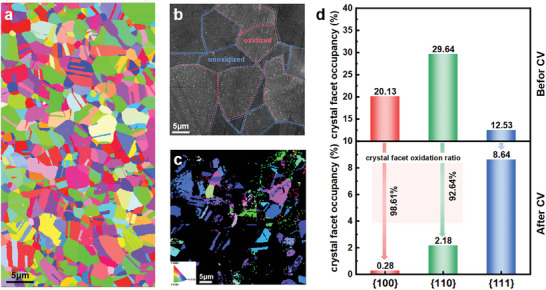
Catalytic activity of different crystal facets in polycrystals. a) EBSD of pristine polycrystal. b) SEM images of the catalytic activity of different crystal facets. c) EBSD images of constructed polycrystals. d) The occupancy ratio of different crystal facets before and after reconstruction.

The IPF coloring of the pristine polycrystal MEA is shown in **Figure** **3**a. The sample is composed of a variety of grains, each with unique orientations. These orientations are evenly distributed, indicating that the sample does not exhibit textures or preferred facets. Subsequently, the SEM image of the sample after OER activation is displayed in Figure 3b. It can be observed that bright spots on the surface of certain grains indicate that these grains are rich in oxides, suggesting their high activity during the OER process. On the contrary, other grains (as marked by blue lines) remain almost unaffected, implying their inactivity in the OER process. This phenomenon is also evident in the elemental distribution mapping (Figure S12, Supporting Information), showcasing varying oxygen concentrations across distinct crystal faces.

Moreover, Figure 3c presents the IPF after activation. It shows the correlation between the orientation of the crystallographic facets and the OER activity. Specifically, the crystal facets exhibiting lower OER activity maintained their pristine states. On the other hand, the highly active crystalline surfaces exhibit significant structural changes, mainly in the form of being covered by oxides or appearing amorphous. We note that the presence of oxide coverage (due to OER) prevents the identification of specific facets through IPF signals (black part). Therefore, most of the {100} facets are not observed (red coloring part), while a significant number of {111} facets (blue coloring part) and some {110} facets (green coloring) are still present. These results further validate the findings based on single crystals, the {100} facets are more active for OER for FeCoNiCr MEAs.

### 2.3 Mechanism Study

**Figure 4:**
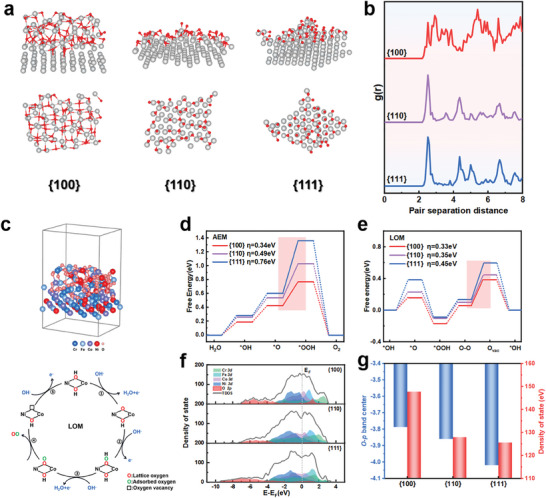
DFT calculation. a) The oxidation degree on the single crystal surface. b) Radial distribution function. c) Structural modeling of {100}and schematic illustration of LOM pathway. d) The Gibbs free energy diagrams of OER of the AEM pathway. e) The Gibbs free energy diagrams of OER of the LOM pathway. f) Total density of states and projected density of states. g) d‐band center and total density of states at the Fermi level.

We carried out density‐functional theory (DFT) to reveal the significant differences in the catalytic activity of different crystal facets of FeCoNiCr MEAs. We constructed model structures of FeCoNiCr MEAs with different crystalline oxides as the active phase structure for OER catalysis. Particularly, we find that the {100} facets exhibit a more pronounced tendency toward amorphous‐crystal compared to the {110} and {111} facets (**Figure** **4**a).

We computed the in‐plane radial distribution function (RDF) in Figure 4b. The RDF peak for the reconstructed {100} facets is broader, indicating the more uniform distribution of atomic distances and emphasizing its amorphous characteristics. In contrast, the reconstructed {110} and {111} facets exhibit sharper RDF peaks, suggesting a more regular distribution of atomic distances, indicating that they experience less surface reconstruction, consistent with the conclusions drawn from the electrochemical tests mentioned above.

We attribute that the reduced in‐plane density of the {100} facets could be the underlying origin. Since the {100} facet possesses low in‐facet density compared to {110} and {111} facets, this makes it easier to bond with oxygen on the surface. Furthermore, the larger distance of neighboring atoms in the {100} in‐plane direction can lead to an increase in surface defects. These defects play a crucial role in catalytic reactions as they provide regions with incomplete atomic structures, aiding the progress of catalytic reactions.

DFT calculations aimed at investigating the four‐electron steps involved in the OER, with a detailed focus on the reaction mechanisms on the reconstructed {100}, {110}, and {111} facets. Along the OER pathway, we considered both the traditional adsorbate evolution mechanism (AEM) and the lattice oxygen oxidation mechanism (LOM). Initially, we systematically calculated the overpotential for potential adsorption sites of Ni, Fe, and Co on the reconstructed {100} facets, serving as reaction intermediates (see Figure S14, Supporting Information). The results indicated significantly higher activity for Ni and Co sites compared to Fe. Therefore, we selected Ni and Co sites as the primary adsorption sites for intermediates.

As shown in Figure 4d,e, the LOM mechanism exhibited lower theoretical overpotentials compared to the AEM. In the mechanism calculation of LOM, the rate‐determining step (RDS) corresponds to the desorption of oxygen, and the theoretical overpotentials for the three crystal facets are 0.33, 0.35, and 0.45 eV, respectively. This trend is consistent with experimental results.

Meanwhile, the total density of states on the reconstructed {100} facet significantly increases near the Fermi level in comparison to the reconstructed {110} and {111} facets (see Figure 4f). This indicates that this reconstructed surface provides more electronic states, which helps enhance the kinetics of the reaction.

A useful descriptor of oxygen activity is the distance between the O 2p band center and the Fermi level. It has been reported that a sufficiently high O 2p band center is required to facilitate lattice oxygen release.^[^ ^44^, ^45^ ^]^ The upward shift of the O 2p band results in deeper penetration of the Fermi level into the O 2p band, further promoting electron flow from oxygen sites at anodic potentials and facilitating the release of lattice oxygen.^[^ ^46^, ^47^, ^48^, ^49^ ^]^ Therefore, as shown in Figure 4g, the {100} facets, with a higher O 2p band center, enhance the formation of lattice oxygen, thus promoting the LOM. Furthermore, the hybridization between the metal and the oxygen is more pronounced on the reconstructed {100} facets, aiding electron transfer from metals to oxygen. This electron transfer can enhance the reaction rate through the hybridization effect. Subsequently, as shown in Figure 4g, we computed the positions of the O 2p band center on the {100}, {110}, and {111} facets. The O 2p band center for the {100}, {110}, and {111} facets are −3.78, −3.86, and −4.02 eV, respectively. The O 2p band exhibits a noticeable shift toward the Fermi level. The O 2p band closer to the Fermi level on the {100} facet enhances the formation of lattice oxygen, improving reactivity and efficiency. Additionally, the Fermi level can penetrate deeper into the O 2p band center, promoting the release of oxygen.^[^ ^8^ ^]^ This result is also confirmed by the higher lattice oxygen content in the {100} facets as analyzed by O 1s XPS (Figure S18, Supporting Information).

**Figure 5:**
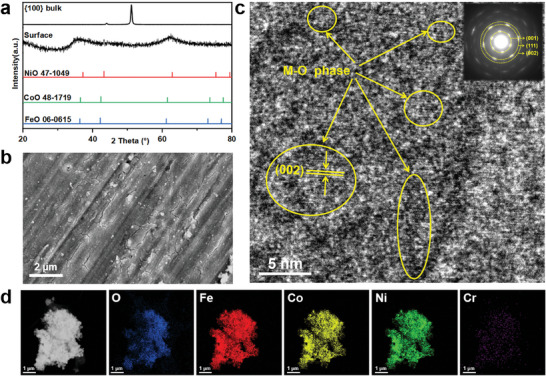
Microstructural analysis on the reconstructed surface {100}. a) XRD pattern of the metal oxide stripped from the surface of the block. b) SEM image of the reconstructed surface. c) HRTEM image of the metal oxide, inset with SAED pattern. d) TEM image of the metal oxide and corresponding elemental mapping image.

To experimentally validate the above insights from DFT calculations, we focused on the in situ growth of oxides on the {100} facets of the MEAs (which showed the best performance in this work) after OER testing. We scraped off the oxide from the surface to remove the interference of the matrix. As shown in **Figure** **5**a, the XRD pattern reveals the diffraction peaks of multi‐metal oxides corresponding to FeO (JCPDS 06–0615), CoO (JCPDS 48–1719), and NiO (JCPDS 47–1049). The weak diffraction peak intensity suggests that the reconstructed materials are mostly amorphous, which is consistent with our DFT calculations. In Figure 5b, the spots of the M─O phase (M = Fe or Co or Ni) are shown, but the details of the chemical nature of M could not be discerned due to their similarity. Further structural characteristics of the oxides at higher resolution were obtained by TEM. As shown in Figure 5c, the selected area electron diffraction (SAED) pattern indicates an amorphous‐crystal hybrid structure. The elemental mapping image in Figure 5d shows the uniform distribution of Fe, Co, Ni, and O throughout the oxide. This hybrid structure integrates the high electrical conductivity of crystalline materials with the unique local coordination characteristics of amorphous materials, effectively facilitating the adsorption and desorption processes of molecular intermediates.^[^ ^50^, ^51^, ^52^ ^]^

### 2.4 Detecting the OER Intermediates

Previous studies have demonstrated that alcohol molecules, acting as nucleophilic reagents, readily undergo reactions with electrophilic OH^*^ species adsorbed on the OER electrode surface.^[^ ^53^ ^]^ Consequently, in the context of mixed electrolytes containing methanol and potassium hydroxide, anodic oxidation exhibits competition between the methanol oxidation reaction (MOR) and OER, which facilitates the detection of the first OER intermediate OH^*^. This competition mechanism aids in monitoring the OER process.

**Figure 6:**
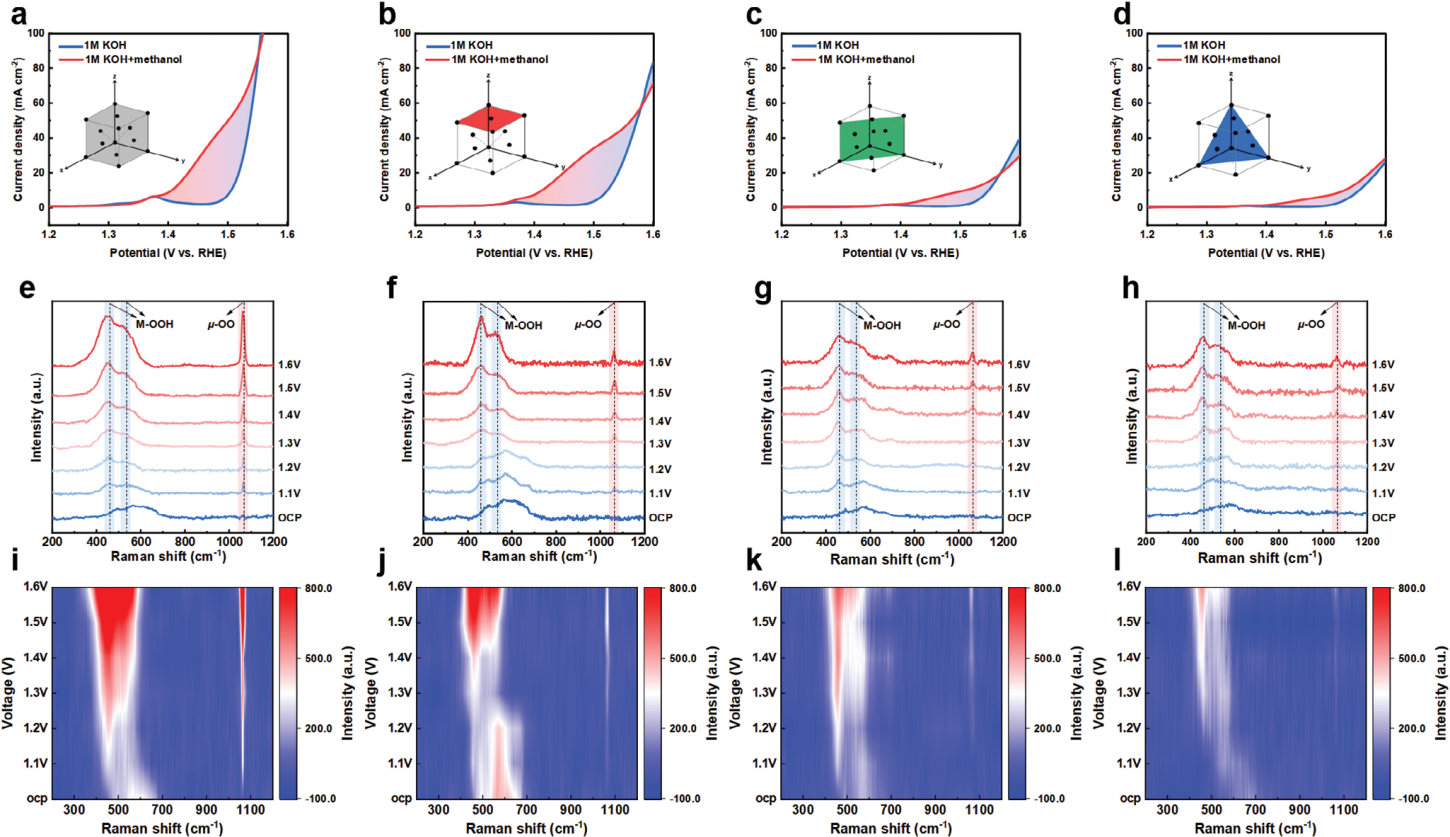
The electrochemical activity mechanism. Polarization curves of a) polycrystal, b) {100} facets, c) {110} facets, and d) {111} facets of FeCoNiCr in 1 m KOH with and without methanol. In situ Raman spectra of e) and i) polycrystal, f) and j) {100} facets, g) and k) {110} facets, h) and l) {111} facets of FeCoNiCr.

As shown in **Figure** **6**a–d, with increasing voltage, the MOR current density is more sensitive to both polycrystal and single crystal {100} facets, and higher adsorption of OH^*^ on the reconstructed {100} facets. In contrast, the reconstructed {110} and {111} exhibit minimal ^*^OH adsorption. The adsorption energies of OH^*^ on different crystal facets were compared in addition to the DFT calculations (Figure 4e). The results indicate that OH^*^ adsorption energy on the {100} facets is significantly lower than that on the {110} and {111} facets and has a higher affinity in the adsorption process of OH^*^.

We conducted In situ Raman measurements to monitor the intermediate active species during OER, and the corresponding results are shown in Figure 6e–h. Within the 400–600 cm^−1^ range, a camel‐back‐shaped peak is observed at open‐circuit potential and low voltages. This peak can be divided into three distinct characteristic peaks. Among these, a pair of M─O Raman signals at 465 and 540 cm^−1^ reflects the bending and stretching vibration modes of M─OOH. The peak at 590 cm^−1^ corresponds to vibrations of M─O bonds within M─OH.^[^ ^54^, ^55^ ^]^ Additionally, a voltage‐dependent Raman peak is observed at 1064cm^−1^, referred to as µ‐OO.^[^ ^56^ ^]^ The appearance of the µ‐OO peak is direct evidence of lattice oxygen involvement in the reaction, which proves that the FeCoNiCr MEAs with different crystal facets follow LOM rather than AEM, which is also in agreement with the results of the DFT calculations mentioned above. Variations in Raman spectra among polycrystal and different single‐crystal facets of FeCoNiCr are primarily manifested in the peak intensities and the µ‐OO peak. Evidently, the peak intensities in the polycrystal and {100} facets are notably higher compared to those in {110} and {111} facets. Since alterations in peak intensity signify the advancement of the reaction and the catalyst's activity, this observation indicates stronger adsorption of intermediates and superior catalytic performance in the polycrystal and {100} facets. Furthermore, µ‐OO can directly reflect the generation of intermediates during the catalytic reaction. Lower voltages favor the production of µ‐OO, making it more advantageous for the reaction. polycrystal samples exhibit µ‐OO presence from open‐circuit voltage, while {100} facets manifest it at 1.1 V. In contrast, {110} and {111} facets require higher voltages to drive the formation of µ‐OO.

We introduced the tetramethylammonium cation (TMA+) as a chemical probe (Figure S17, Supporting Information) into the solution because of its specific interaction with negative oxygenated species. It can be seen that after the addition of TMA+, µ‐OO is weakened and accompanied by the appearance of the characteristic TMA+ peaks, originating from the aggregation of TMA+ and O^2−^, and subsequently the original LOM has changed and the reaction proceeds along the AEM.

The high‐resolution XPS fitted spectra for polycrystal and single crystals with three distinct facets following OER activation (Figure S18, Supporting Information). The spectra were analyzed considering Fe 2p, Co 2p, Ni 2p, and satellite peaks, respectively. The variations observed among these samples are minimal, indicating that their reconstructions are of the same nature.

## 3 Discussion

The above combined results demonstrate that the {100} facets of the FeCoNiCr MEA are more active toward OER, which is related to the formation of an amorphous reconstructed surface. These findings are consistent with the fact that polycrystal materials have better activity, as shown in Figure 2. Previous results also indicate that the grain boundaries of the polycrystal MEA are easier to reconstruct and amorphized.^[^ ^14^, ^57^ ^]^ In that work, a logarithmic dependence of OER performance on mean grain size was observed, spanning three orders of magnitude, as measured by evaluators such as overpotential and Tafel slope.^[^ ^14^ ^]^ Spatially resolved microscopic imaging of activated samples indicates that the GBs undergo substantial reconstructions and are enriched with in situ formed metal oxides (M─O, M = Fe, Co, Ni) and amorphous regions.^[^ ^14^ ^]^

These findings bring us the hope that a variety of methods can be employed to enhance surface reconstruction and amorphous formation, ultimately enhancing the activity of MEAs for OER. 1) Modifying crystal stability: For instance, inducing atomic strain to expand the lattice distance, particularly at metal‐oxide interfaces.^[^ ^15^ ^]^ 2) Designing compositions to promote metallic glass formation: for example, compositions with significant differences in atomic sizes and negative heat of mixing among the main constituent elements could facilitate reconstruction and amorphization.^[^ ^58^ ^]^ 3) Non‐equilibrium preparation methods: Utilizing techniques like rapid quenching and vapor deposition to promote either the formation of amorphous structures or the crystalline defects that help the surface reconstructions.^[^ ^59^ ^]^

## 4 Experimental Section

### Synthesis of Single Crystals and Polycrystal Electrodes

In the initial step, the investigated FeCoNiCr single crystal alloy with a basic composition of equi‐atomic ratio. A single crystal bar, 14.00 mm in diameter and 169.80 mm in length was solidified using a modified Bridgman directional casting furnace by spiral grain selection at the Beijing Institute of Aeronautical Materials. The growth orientation of single‐crystal bar was determined by Laue diffraction and is along [001] within 5°, a single crystal of MEAs was meticulously acquired.^[^ ^60^ ^]^ Subsequently, a suitable specimen was extracted from the single crystal. Following the initial extraction, the crystallographic orientation of the newly obtained crystal facets was rigorously assessed. This iterative process was performed multiple times to yield crystal facets bearing orientations that most closely approximated {110} and {111}.

Further refinement was carried out on these newly acquired crystal facets to guarantee optimal surface flatness and quality. This entailed a series of procedures including meticulous polishing, precision etching, and the implementation of specialized processing techniques.

Polycrystal MEAs were synthesized solely through the arc melting method, without any additional physical or chemical treatment. The chosen alloying materials (Fe, Co, Ni, Cr) had a purity level surpassing 99.98 wt%. Before melting, the surface oxide layers of the raw materials were removed, followed by ultrasonic cleaning in anhydrous ethanol to eliminate surface impurities.

For the raw materials, weighing a total of 30 g, magnetic stirring was introduced during the arc melting process to ensure thorough mixing and agitation of the materials. At the end of the melting process, the molten alloy was cooled to room temperature in a copper crucible inside the arc melting furnace. MEAs were subjected to electrical discharge machining to dimensions of 1mm × 5mm × 8 mm, subsequently, the samples were subjected to grinding treatment. The grinding process is accomplished using SiC sandpaper. Specifically, the process proceeds as follows: first, rough grinding is performed using 100# grit to remove the surface oxide layer. Subsequently, fine grinding is conducted using 200#, 400#, 800#, 1200#, and 2000# grit sequentially to eliminate surface residual stress layers. Water is employed as a medium in each grinding step, followed by surface polishing with sandpaper until smooth, they were ultrasonically cleaned in anhydrous ethanol. This approach carried out with rigorous scientific methodology, ensured the quality and consistency of the synthesized MEAs.

### Structural Characterization

X‐ray diffraction measurements were performed using a D2 Phaser X‐ray diffractometer manufactured by Bruker, the instrument was fitted with Cu Kα (λ = 1.5406 Å). The Thermo Fisher Scios 2 dual‐beam focused ion beam scanning electron microscope (FIB‐SEM) equipped with an Energy‐Dispersive X‐ray Spectrometer (EDS) was employed to analyze the microstructural morphology and elemental distribution of the sample. The Zeiss Gemini SEM 300 electron backscatter diffraction (EBSD) system was utilized for quantitative and qualitative analysis of crystal facets. X‐ray Photoelectron Spectroscopy (XPS) was used to investigate the elemental types and chemical states on the sample's surface.

In situ Raman spectroscopy measurements were carried out using a confocal Raman microscope (Renishaw, in Via Raman spectrometer, United Kingdom). In this study, a laser with a wavelength of 532 nm was utilized, and the measurement range covered 200–2000 cm^−1^, providing an effective means of distinguishing phase composition. Elemental leaching from alkaline electrolyte solutions after electrocatalysis was detected and analyzed by an inductively coupled plasma optical emission spectrometer (ICP‐OES) on a Varian Vista Pro instrument. The surface morphology of the sample via atomic force microscopy measurements by using Bruker Multimode.

### Electrochemical Measurements

The OER tests were conducted using a conventional three‐electrode system on a CHI 660E workstation (Shanghai). The setup comprised the electrocatalysts as the working electrode (0.5mm × 5mm × 8 mm), a Hg/HgO electrode as the reference, and a platinum sheet as the counter electrode. The activation mode of cyclic voltammetry (CV) is achieved by cyclic scanning within a specific voltage range. The voltage range is from −1.5 to 1 V, with a scanning rate of 100 mV s^−1^, and the number of cycles ranges from 300 to 400. Linear scan voltammetry (LSV) was performed in a 1 m KOH solution saturated with oxygen at 25 °C, ranging from 0 to 1 V versus RHE at a scan rate of 5 mV s^−1^. The electrochemical impedance spectroscopy (EIS) was measured within a frequency range of 100 kHz–0.1 Hz at an amplitude of 5 mV. The electrochemical surface area (ECSA) was calculated using the double‐layer capacitance (C_dl_) method, employing the formula ECSA = C_dl_/C_s_, where C_s_ denotes the specific capacitance of the sample. C_dl_ values for the electrocatalysts were obtained by cyclic voltammetry (CV) at varying scan rates in a non‐faradaic region. All potentials were normalized to the reversible hydrogen electrode (RHE) based on the Nernst equation: E_RHE_ = E_Hg/HgO_ + 0.059*pH + 0.098.

### First‐Principle Calculations

The simulations were conducted using the Vienna ab initio Simulation Package (VASP) employing a spin‐polarized density functional augmented with the Hubbard model (DFT+U approach).^[^ ^61^, ^62^, ^63^, ^64^ ^]^ Interactions between core and valence electrons were modeled using the Projector Augmented Wave (PAW) method alongside the Perdew–Burke–Ernzerhof (PBE) exchange‐correlation function. The correlation energy values (U) for the 3d orbitals of Fe, Co, Ni, and Cr were set to 3.5, 2, 3, and 3 eV. Brillouin zone sampling across all systems was performed using Gamma‐centered Monkhorst‐Pack grids. For the optimization of bulk geometries, a 3 × 3 × 1 Monkhorst‐Pack k‐point configuration was utilized.^[^ ^65^ ^]^ The criteria for convergence of forces and total energies were established at 0.05 eV Å^−1^ and 1 × 10^−5^ eV, respectively.

A four‐layer atomic slab model and optimized the initial unit cell structure using the Monte Carlo method was develpoed. Subsequently, the MEA model was established by employing specific Special Quasirandom Structures (SQS).^[^ ^66^ ^]^ By slicing the MEAs model, single‐crystal facets of {100}, {110}, and {111} were obtained. In the simulation of the catalytic process, considering experimental conditions, the catalyst surface is required to transform into an oxide or hydroxide form, while the metal surface merely serves as a precursor in mimicking the experimental setup. Given that the bottom two layers of the model are pure metals, oxygen atoms were introduced on the catalytic surface. These oxygen atoms relaxed together with the metal atoms of the surface layers, thereby forming a surface oxide layer, to more closely replicate the actual catalytic environment.

## Conflict of Interest

The authors declare no conflict of interest.

## Supporting information

Supporting Information

## Data Availability

The data that support the findings of this study are available in the supplementary material of this article.
