# Supplementary material for: Crystal Facets‐Activity Correlation for Oxygen Evolution Reaction in Compositional Complex Alloys
Source: Adv Sci (Weinh). 2024 Jul 23;11(36):2404095. doi: 10.1002/advs.202404095 (PMC11423224; doi:10.1002/advs.202404095)
Supplement: Supplementary file 1 — Supporting Information [file ADVS-11-2404095-s001.docx]

Supplementary Materials

**Crystal facets-activity correlation for oxygen evolution in compositional complex alloys**

*Hui-Feng Zhao^1†^, Jun-Qing Yao^2†^, Ya-Song Wang^2^, Niu Gao^2^, Tao Zhang^1^, Li li^1^, Yuyao Liu^1^, Zheng-Jie Chen^3^, Jing Peng^3^, Xing-Wang Liu^2*^, Hai-Bin Yu^1^**

*^1^Wuhan National High Magnetic Field Center & School of Physic, Huazhong University of Science and Technology, Wuhan 430074, China*

*^2^State Key Laboratory of Materials Processing and Die and Mould Technology, School of Materials Science and Engineering, Huazhong University of Science and Technology, Wuhan 430074, China*

*^3^Shenzhen Key Laboratory of Energy Materials for Carbon Neutrality, Shenzhen Institutes of Advanced Technology, Chinese Academy of Sciences, Shenzhen, 518055, China*

*^†^These authors contributed equally*

*^*^Corresponding authors (e-mail:* *liuxw@hust.edu.cn; haibinyu@hust.edu.cn)*

Table S1. Comparison of OER performance and characteristics of various single crystal electrocatalysts and the crystal facets influence on OER.

| **Catalysis** | **Active facet** | **Remarks** | **Ref.** |
| --- | --- | --- | --- |
| Fe_2_O_3_ Hematite in 1 M NaOH | (012)and (012)-O | Activity order (012) > (104) >（110);  Dh = 105 mV  No surface reconstruction was reported | [1] |
| MnO manganosite in 0.1 M of KOH | (100) | MnO (100) planes with higher adsorption energy of O species could largely promote the electrocatalytic activity for the OER and the ORR. | [2] |
| Co_3_O_4_ in 1 M KOH | Activity order {111} > {112} > {110} > {001} | the {111} facet possesses the biggest dangling bond density, highest surface energy, and smallest absolute value ofΔG_H*_, leading to the enhanced electrocatalytic energy, and smallest absolute value of ΔG_H*_, leading to the enhanced electrocatalytic performance. | [3] |
| Co_3_O_4_ in 0.1 M KOH | {111} | Attributed to the higher density of cobalt ions on the (111) surface compared to that on the (100) surface | [4] |
| Ni_3_S_2_ in 1 M NaOH | {210} | Low free energy based on computation | [5] |
| CoSe_2_ | (001) |  | [6] |
| Co_3_O_4_-NP in 1 M KOH | (112) |  | [7] |
| RuO_2_ | (100) | No lattice oxygen exchange | [8] |
| CoMoO_4_ | (110) | The calculation of {110} reveal the stronger binding and more stable adsorption states between Co and O for the in‐intermediate *O. | [9] |
| LaCoO_3_ 1MKOH | (100) | Due to the optimal e_g_ electron filling, low free energy of adsorption, and high conductivity | [10] |
| SrRuO_3_ | Activity: SRO(111)>SRO(110)>SRO(001)  stability: SRO(001)>SRO(110)>SRO(111) | The activity is controlled by the surface density of defects (for example, stability of Ru cations) rather than the binding energy between the substrate and oxygenated species. | [11] |
| LaNiO_3−δ_ | Activity: (001)<(110)<(111) facets | the (111)  overpotential is ≈ 30−60 mV lower than for the other facets. While a surface  transformation into oxyhydroxide like NiOO(H) may occur for all three  orientations, it is more pronounced for (111) | [12] |


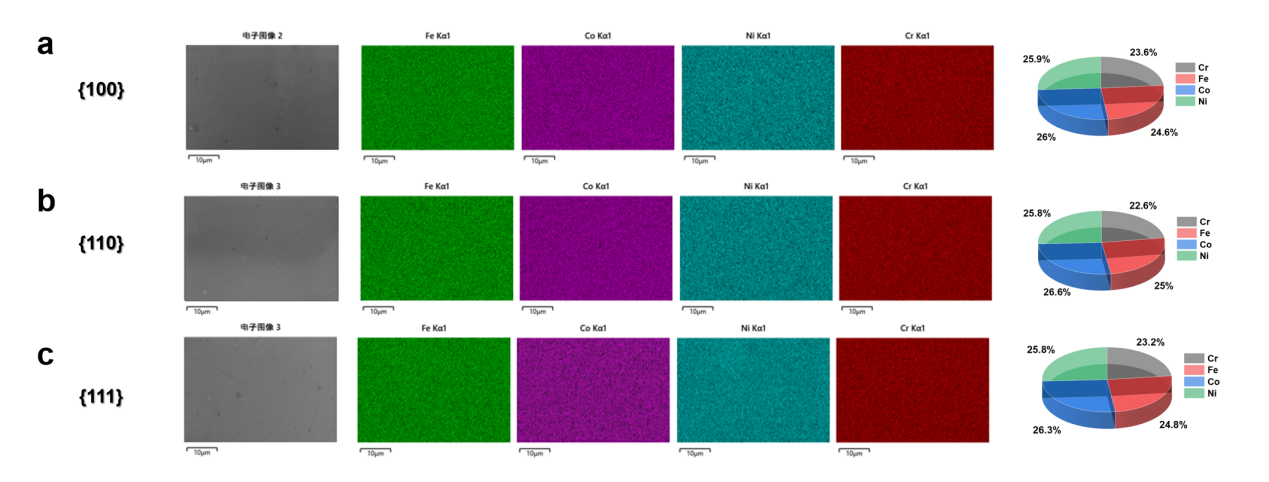


**Figure S1.** **SEM images (top panel) and EDS elemental mapping.** a) {100} facets of FeCoNiCr MEAs, b) {110} facets of FeCoNiCr MEAs, c) {111} facets of FeCoNiCr MEAs.


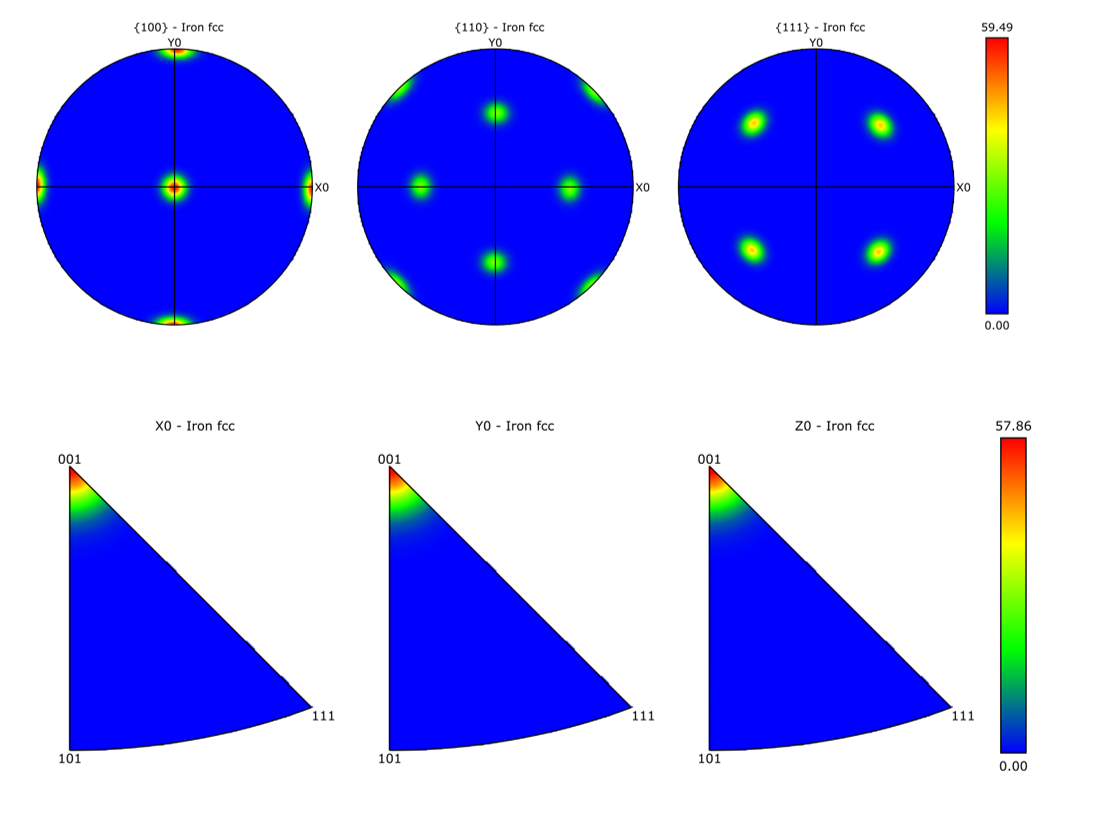


**Figure S2.** Pole figure and inverse pole figure for {100} facets of FeCoNiCr MEAs.


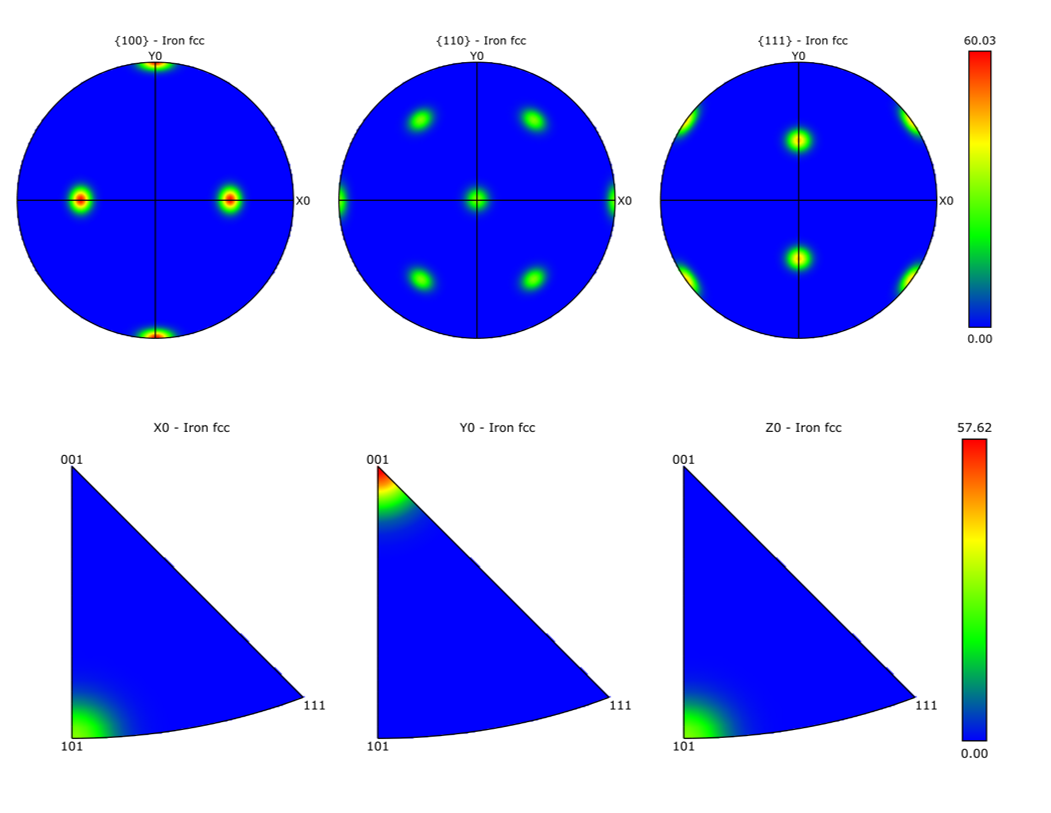


**Figure S3.** Pole figure and inverse pole figure for {110} facets of FeCoNiCr MEAs.


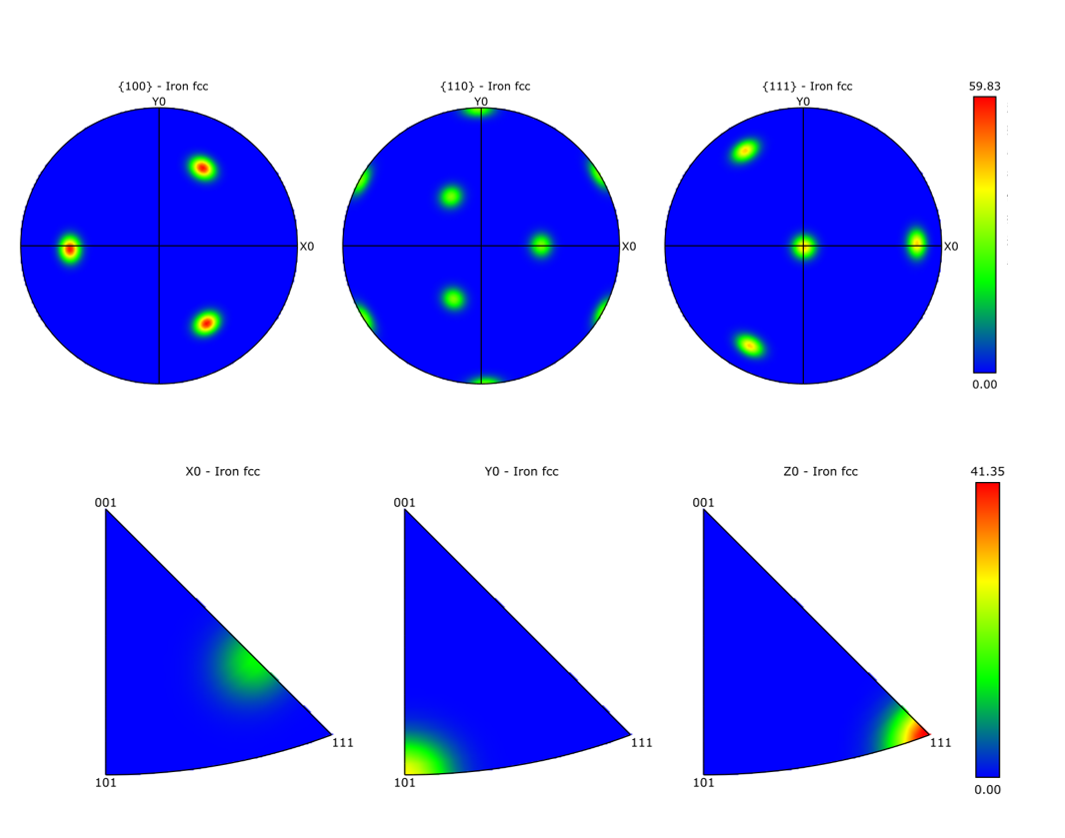


**Figure S4.** Pole figure and inverse pole figure for {111} facets of FeCoNiCr MEAs.


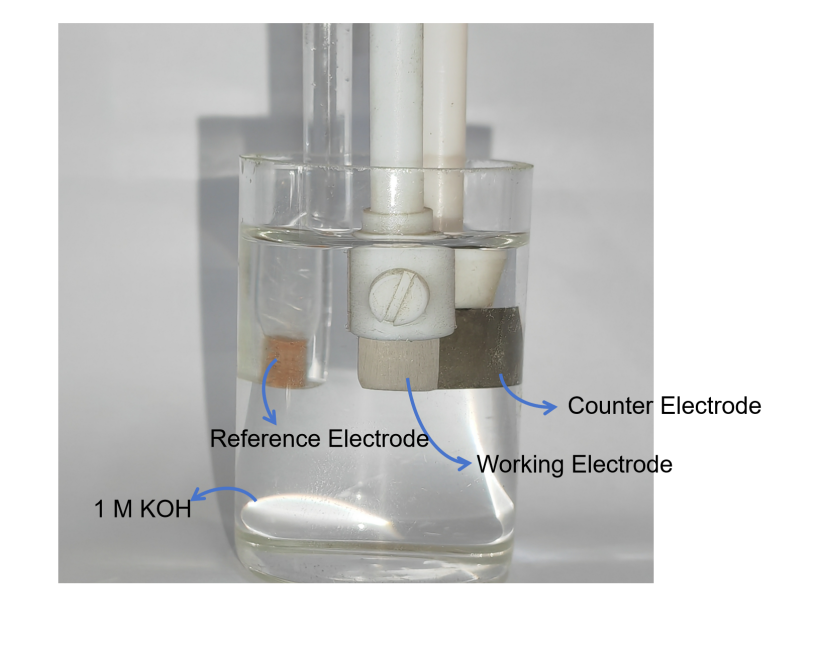


**Figure S5.** **Three-electrode system.**


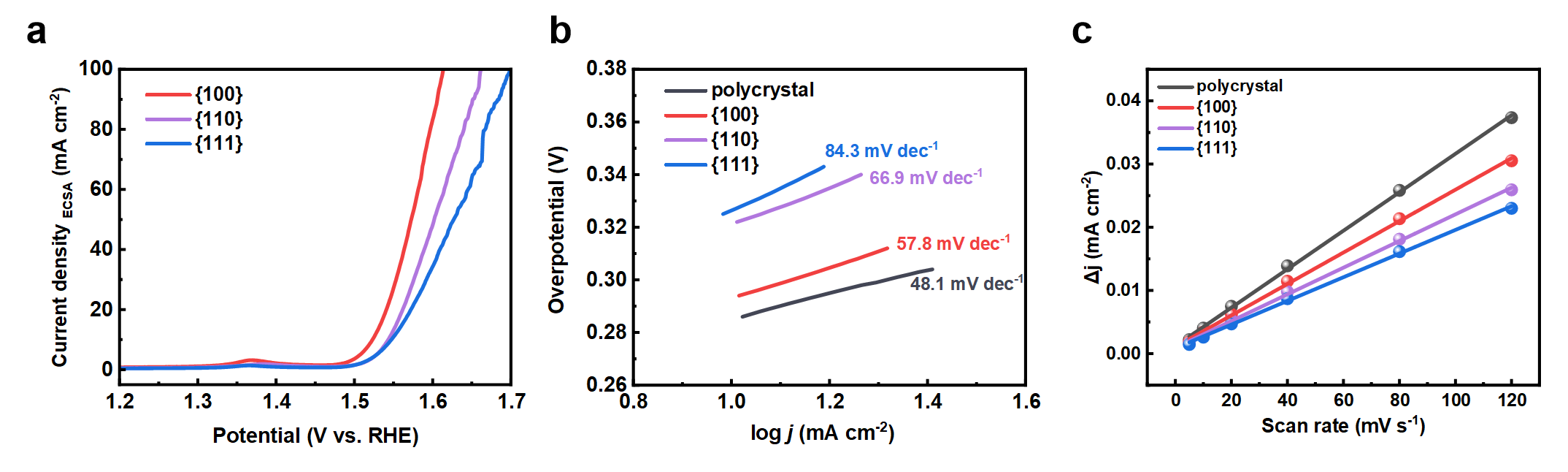


**Figure S6.** **OER performance.** (a) The normalized OER polarization curves, (b) Tafer slopes, (c) Double-layer capacitance C_dl_.


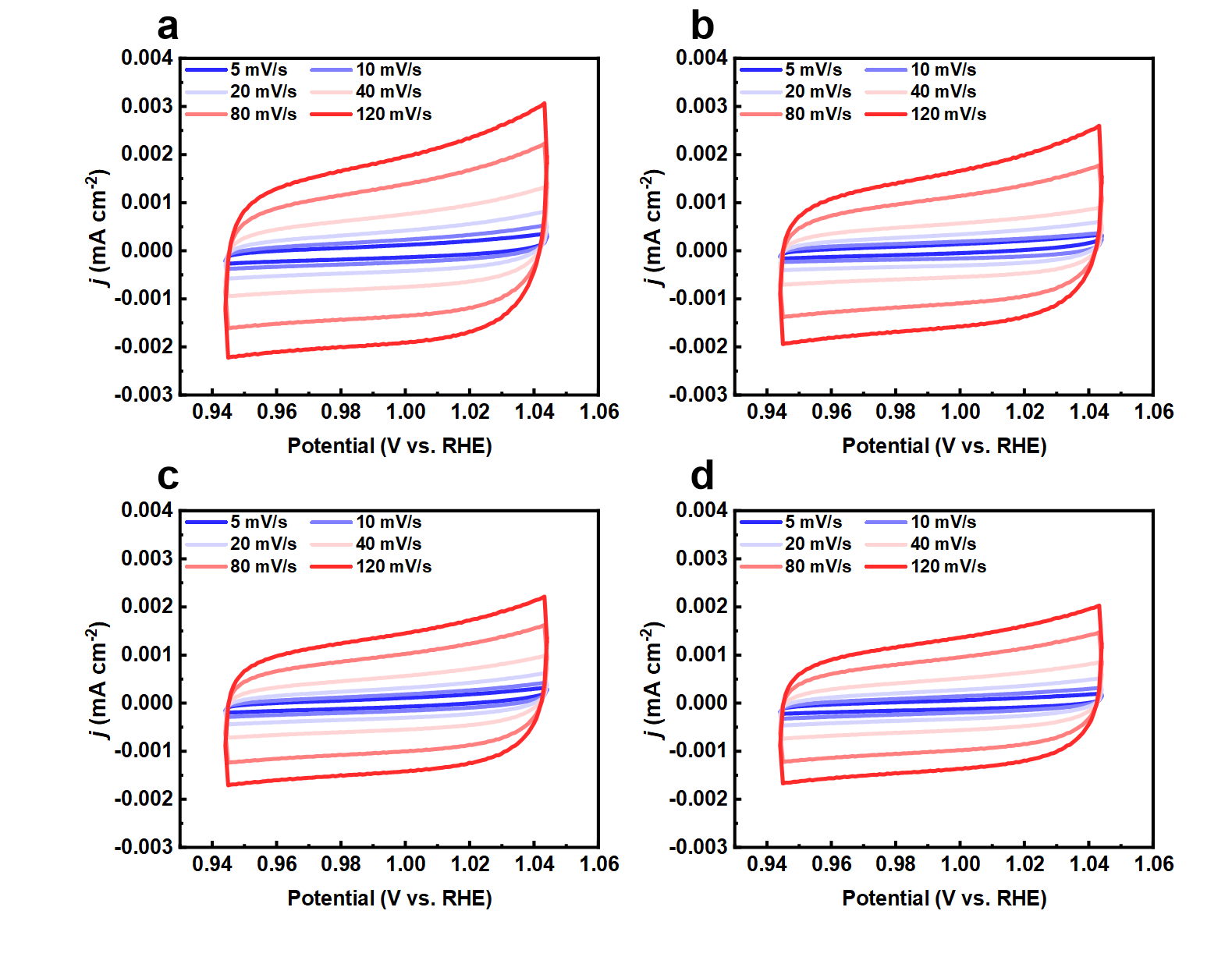


**Figure S7.** ECSA (pristine) of (a) polycrystal of FeCoNiCr MEAs, (b) {100} facets of FeCoNiCr MEAs, (c) {110} facets of FeCoNiCr MEAs, (d) {111} facets of FeCoNiCr MEAs in 1 M KOH at different scan rates (5, 10, 20, 40, 80 and 120 mV/s).


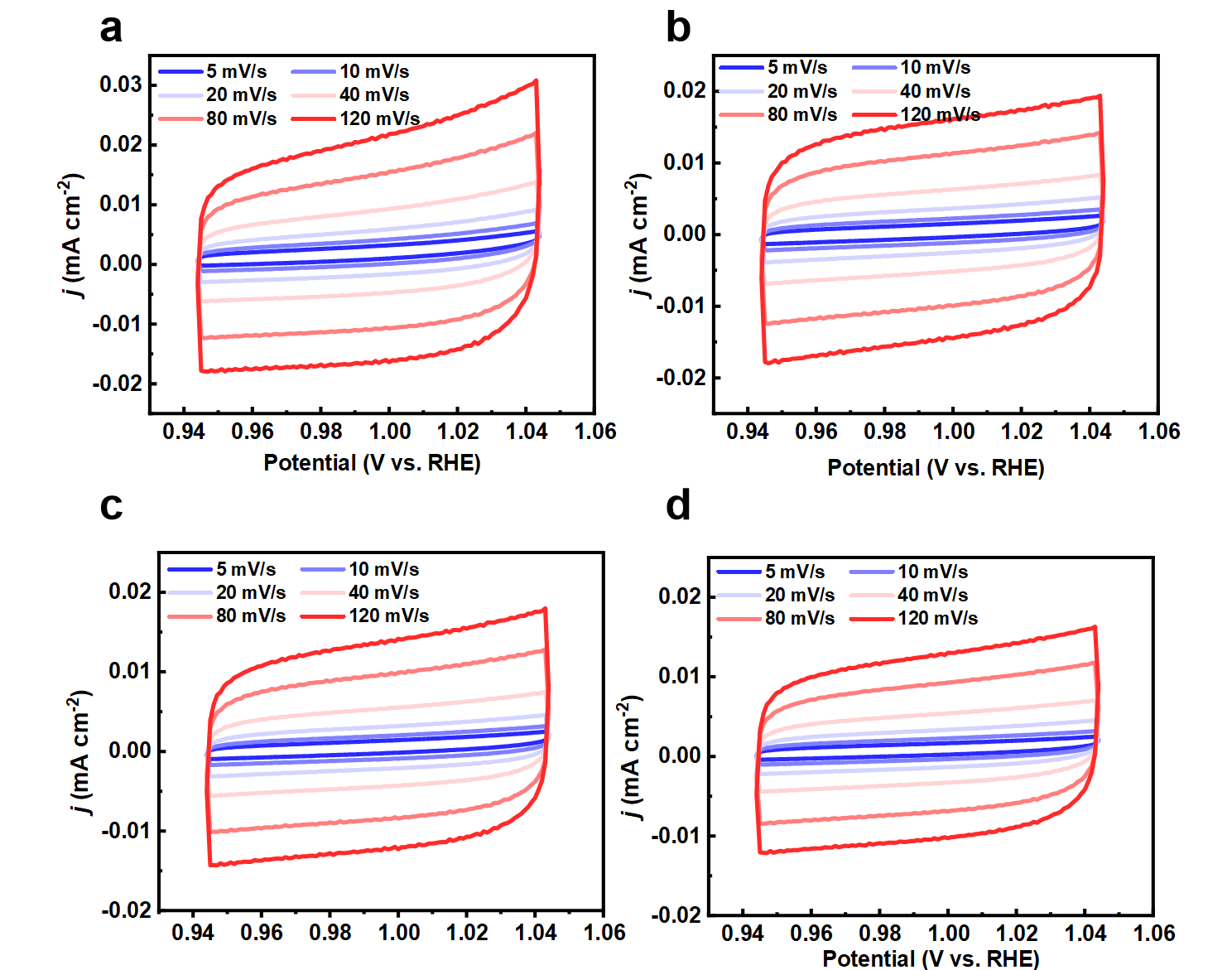


**Figure S8.** ECSA (reconstructed) of (a) polycrystal of FeCoNiCr MEAs, (b) {100} facets of FeCoNiCr MEAs, (c) {110} facets of FeCoNiCr MEAs, (d) {111} facets of FeCoNiCr MEAs in 1 M KOH at different scan rates (5, 10, 20, 40, 80 and 120mV/s).


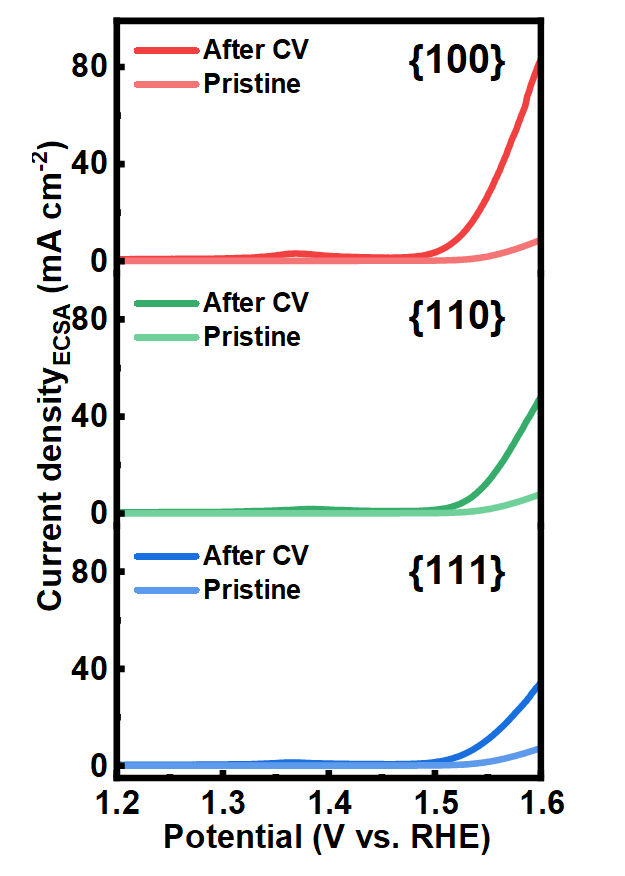


**Figure S9**. The normalized LSV curve of the pristine and reconstructed in 1 M KOH.


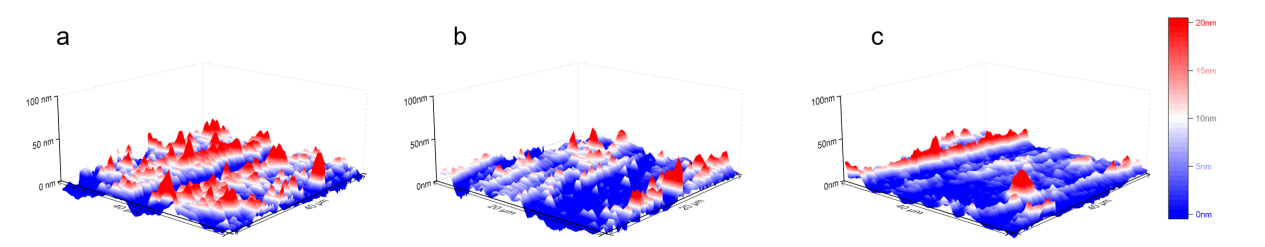


**Figure S10**. Reconstructed surface AFM morphology of a) {100} facets of FeCoNiCr MEAs, b) {110} facets of FeCoNiCr MEAs, c) {111} facets of FeCoNiCr MEAs.


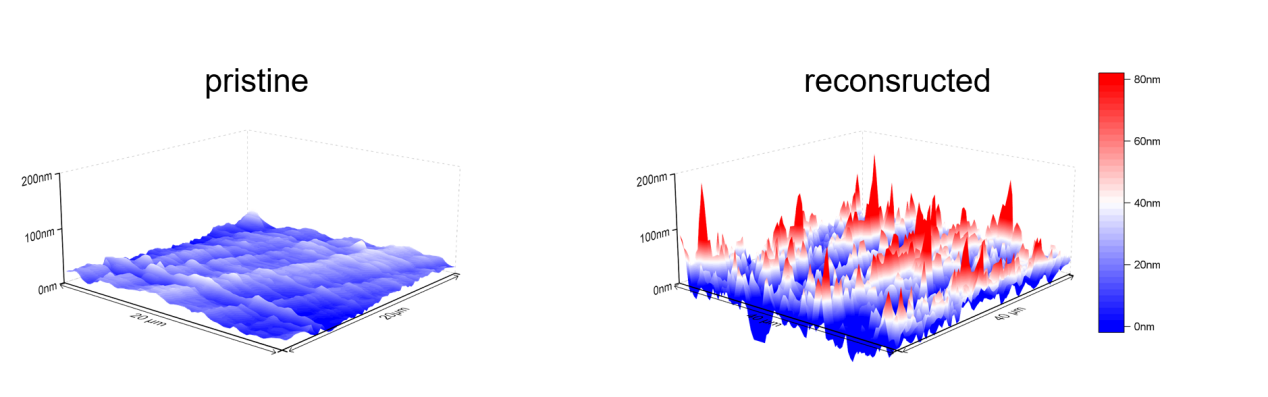


**Figure S11**. Pristine and reconstructed surface AFM morphology of of polycrystal.


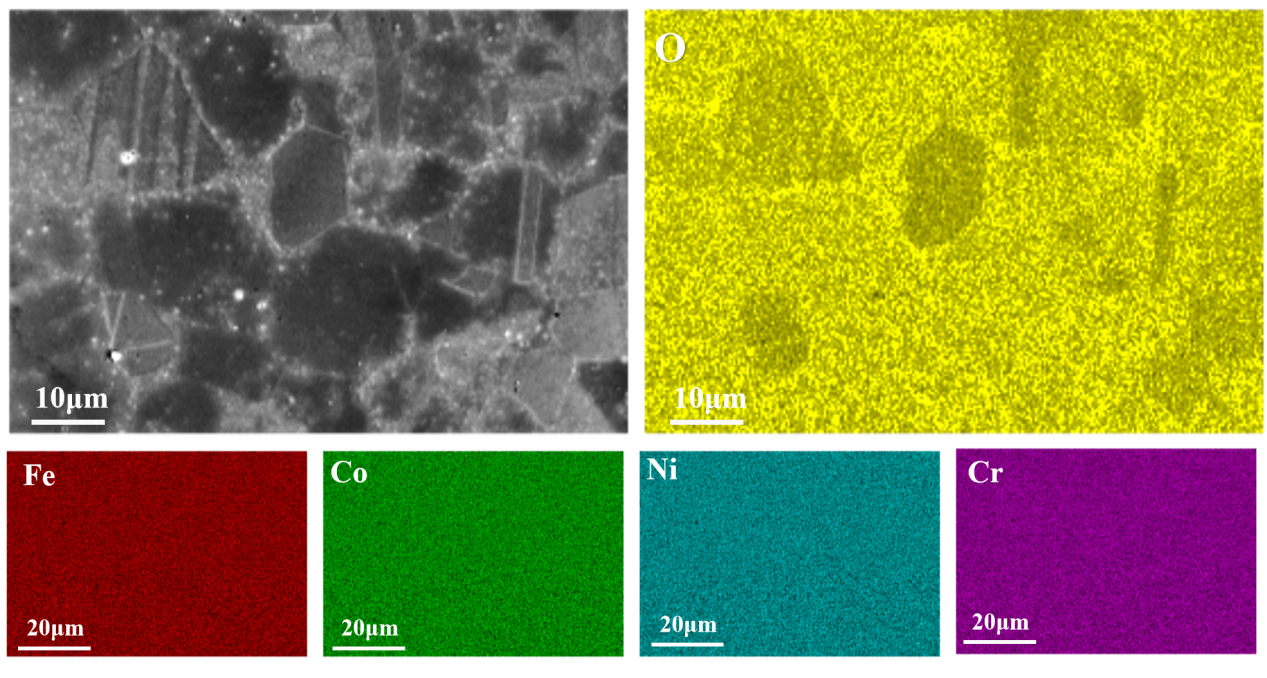


**Figure S12.** SEM image and elemental mapping image of the reconstructed polycrystal.


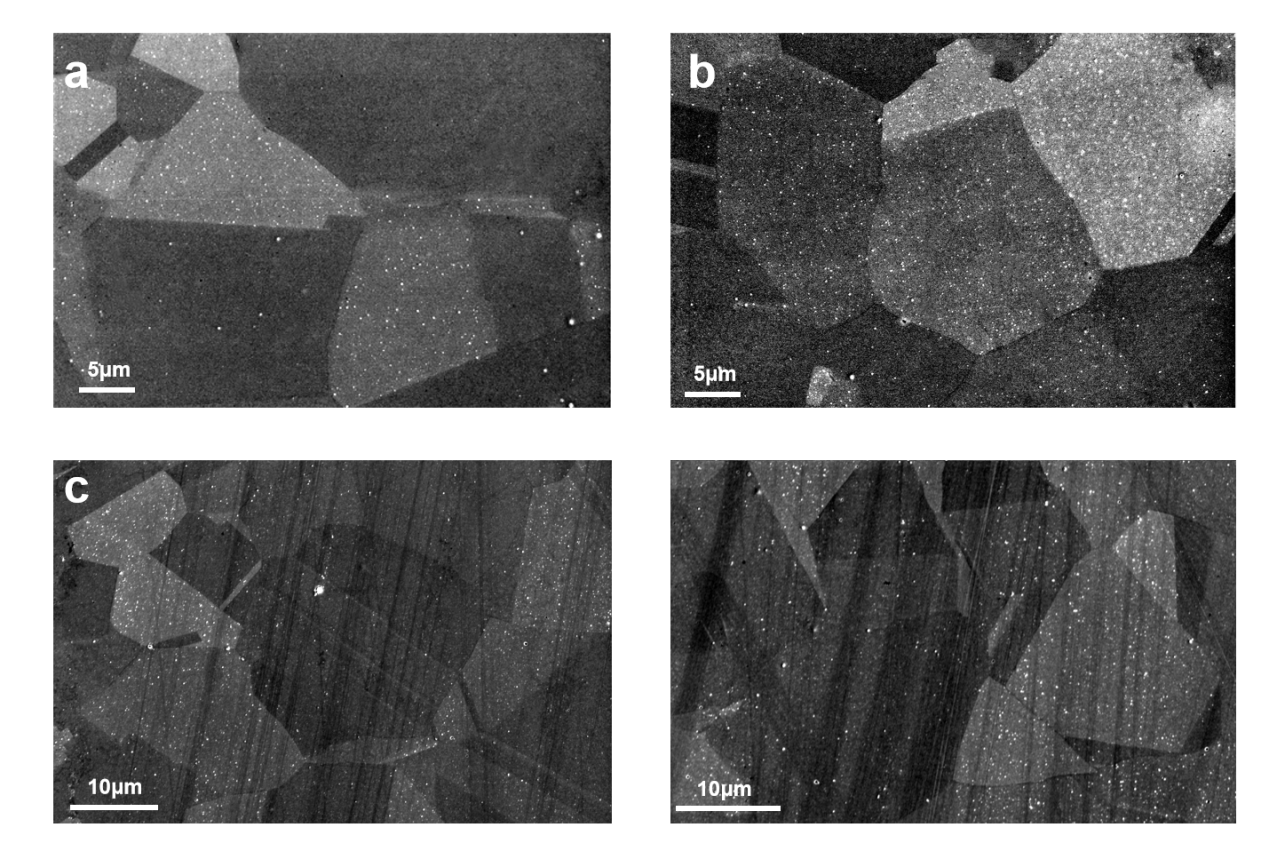
**Figure S13.** SEM image of the reconstructed polycrystal.


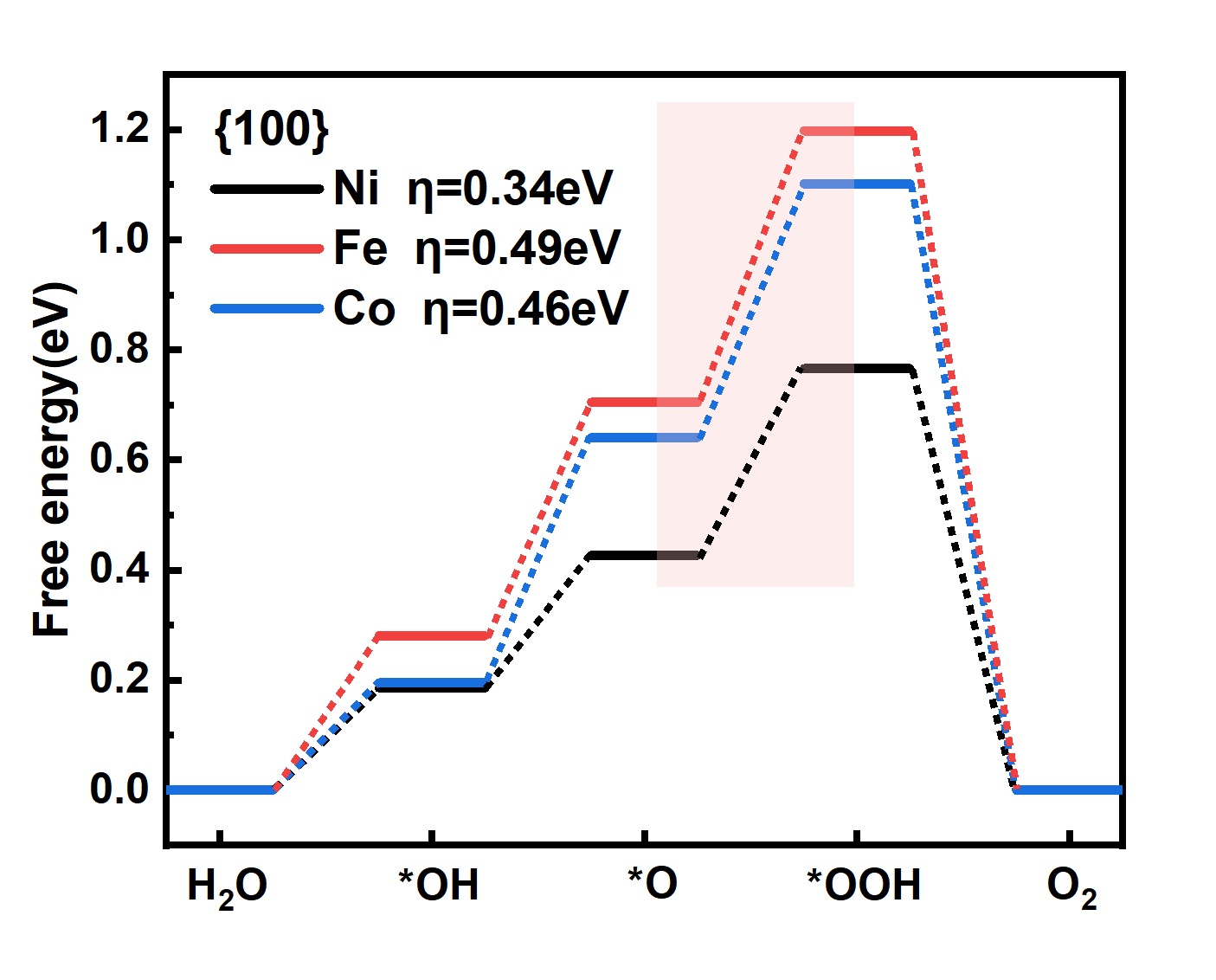


**Figure S14.** The Gibbs free energy diagrams of OER on different site at {100} facets.


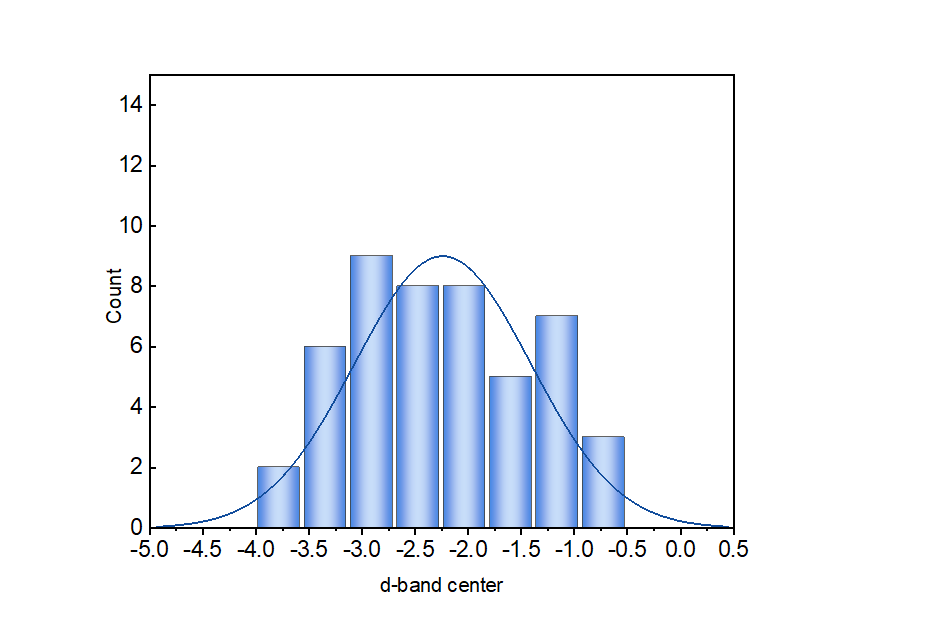


**Figure S15. D-band center of the {100} facets.**


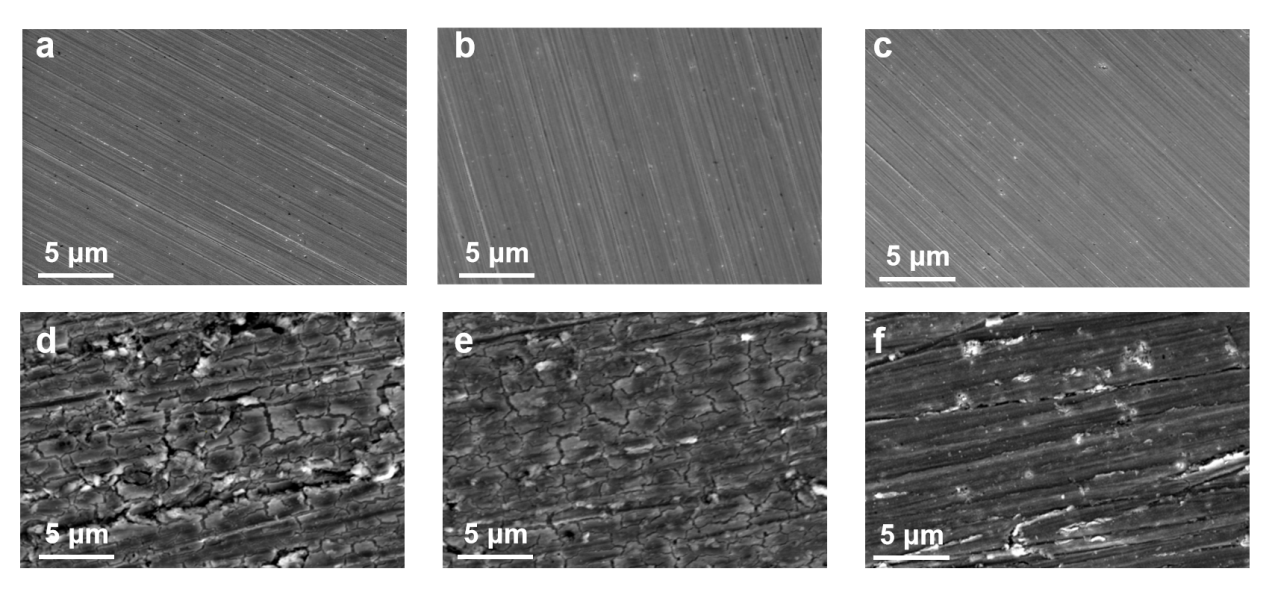


**Figure S16.** SEM image of the pristine surface and reconstructed surface. a-c)Pristine surface of {100}, {110}, {111}. d-f) Reconstructed surface of {100}, {110}, {111} facets .


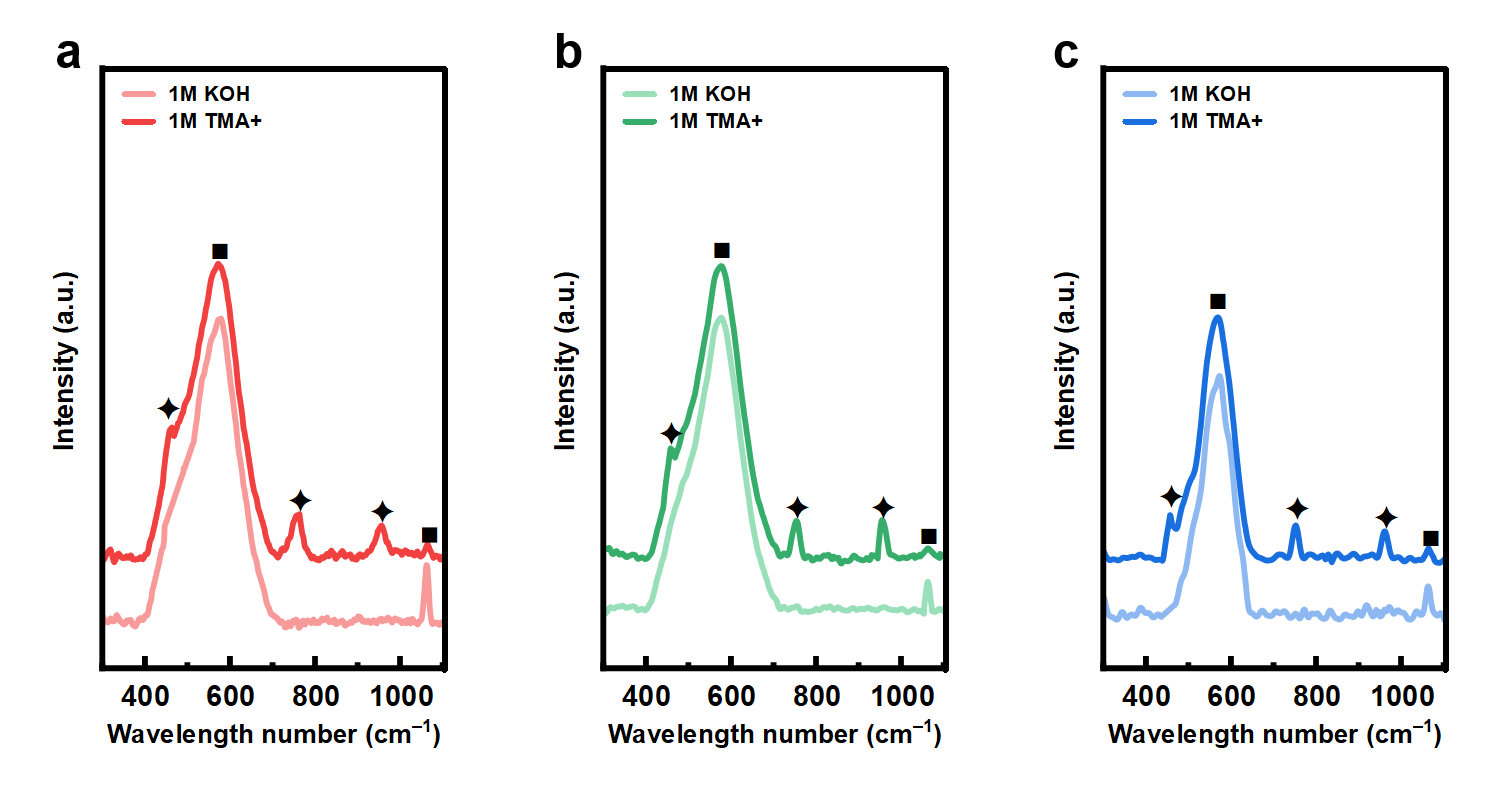


**Figure S17.** Raman spectra at 1 M KOH and 1 M TMA^+^. (a) {100} facets of FeCoNiCr MEAs, (b) {110} facets of FeCoNiCr MEAs, (c) {111} facets of FeCoNiCr MEAs.


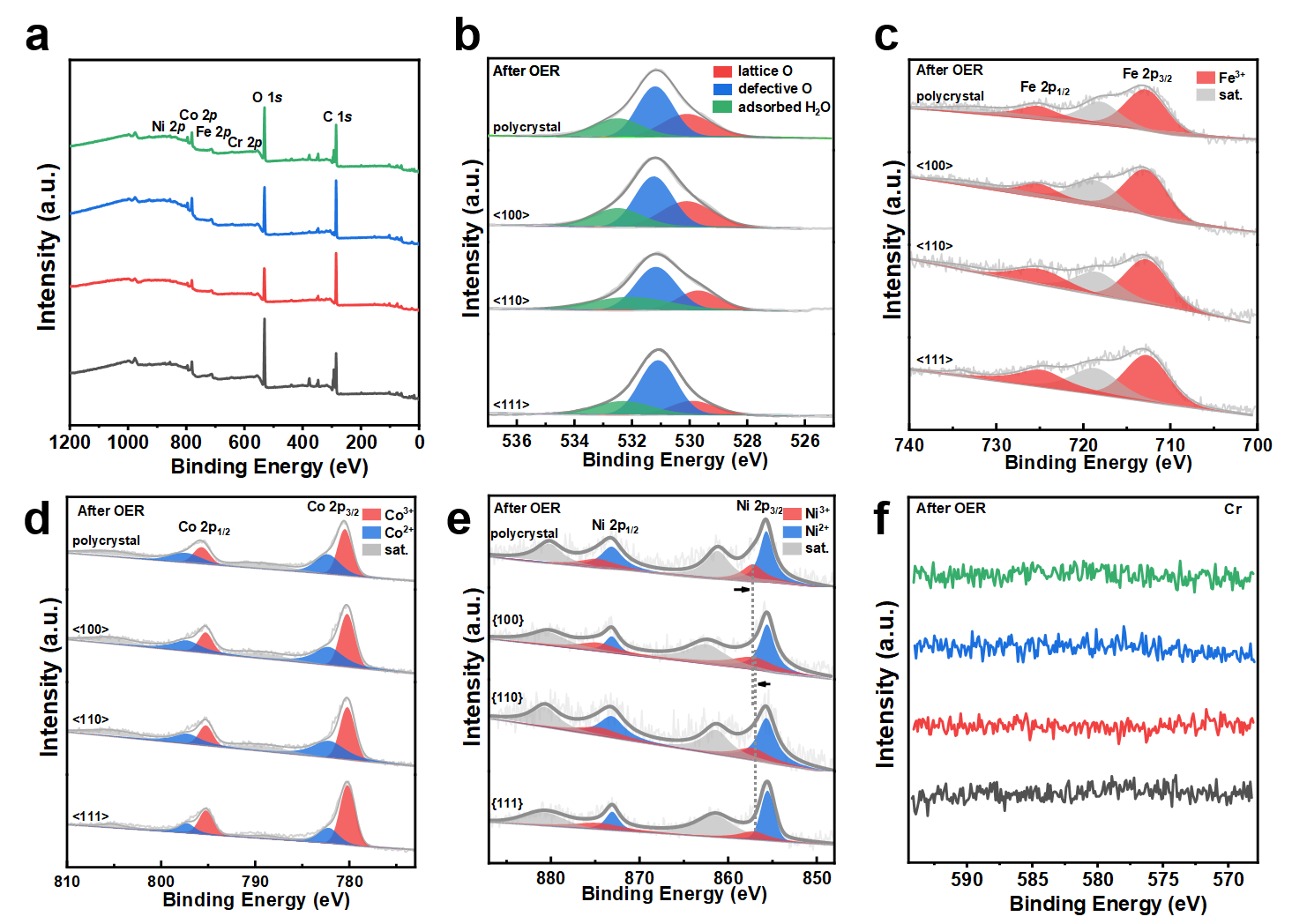


**Figure S18.** (a) XPS survey spectra. High-resolution XPS spectra of (b) O 1s, (c) Fe 2p, (d) Co 2p, (e) Ni 2p, (f)Cr.


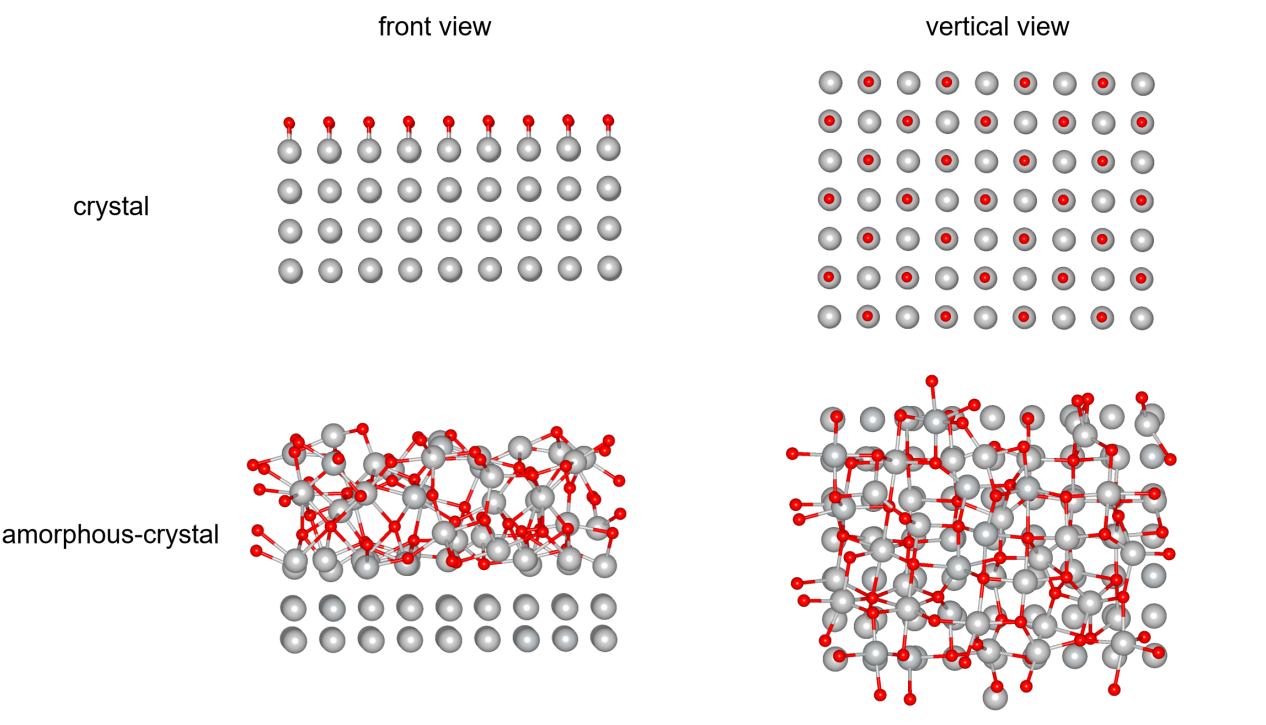


**Figure S19.** Crystal and amorphous-crystal structural modelling of {100} facets.


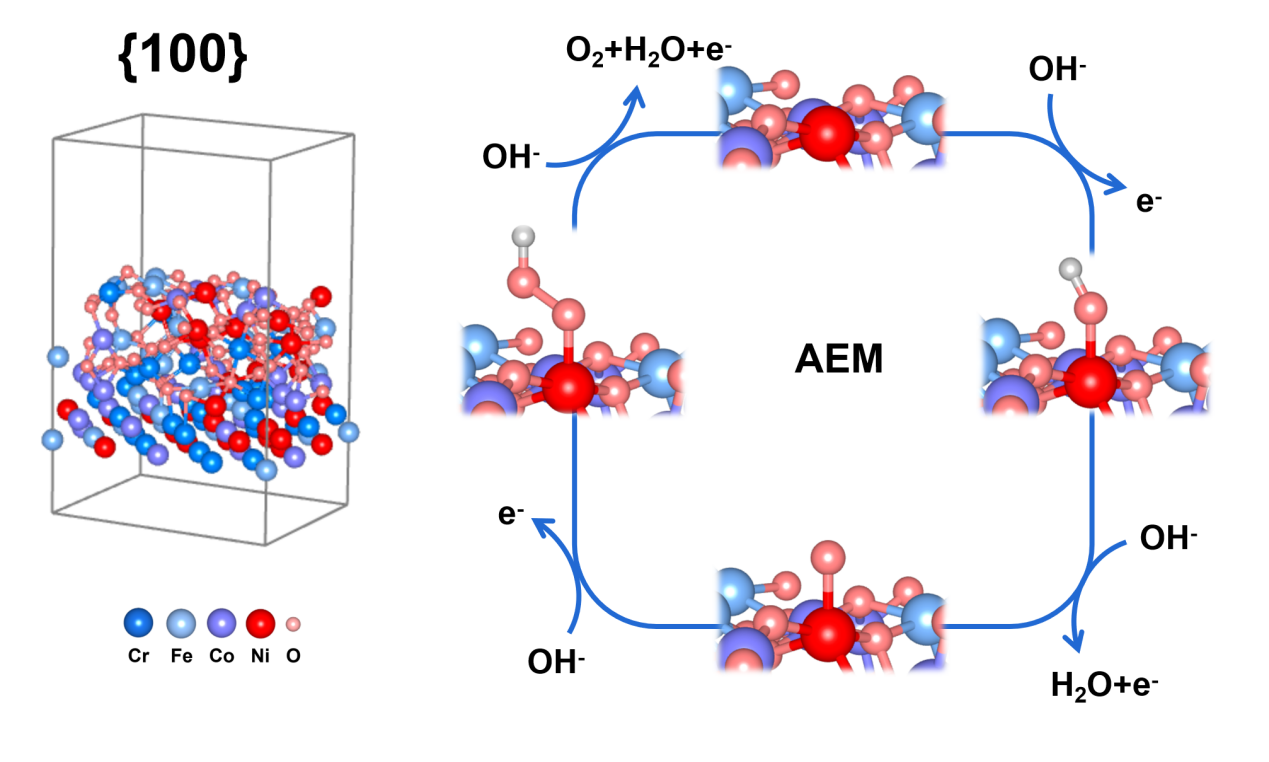


**Figure S20.** Structural modelling and AEM diagrams of {100} facets .


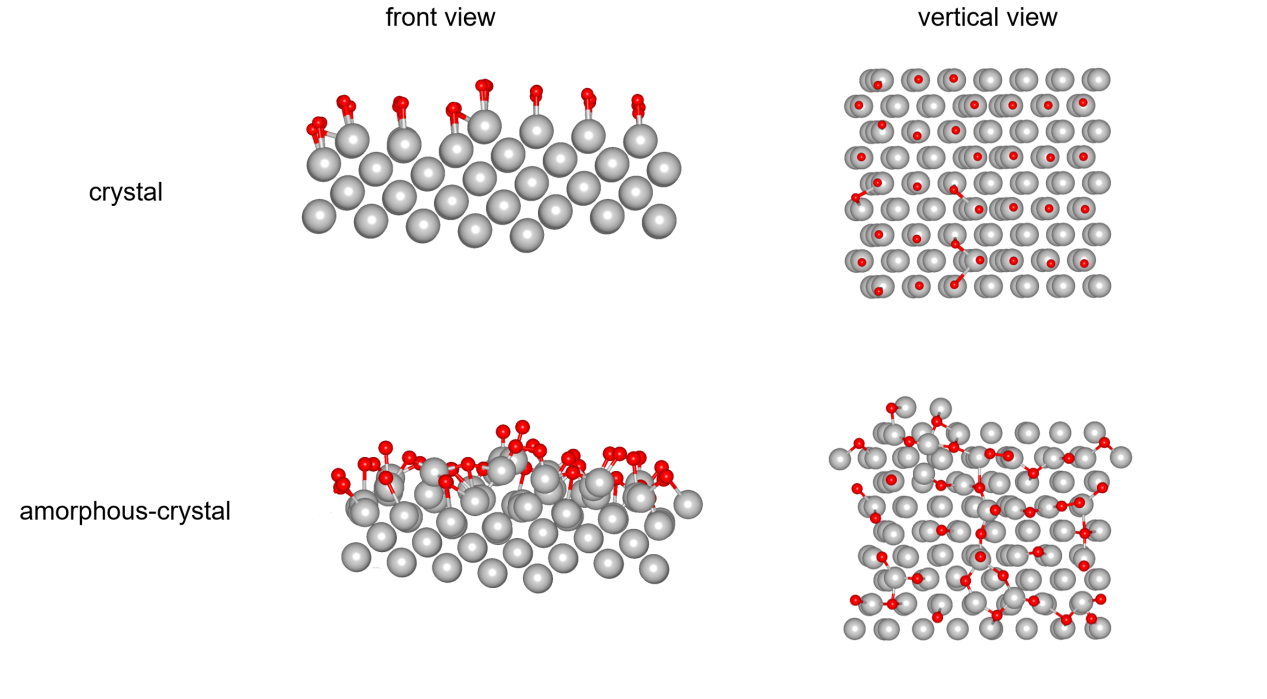


**Figure S21**. Crystal and amorphous-crystal structural modelling of {110} facets.


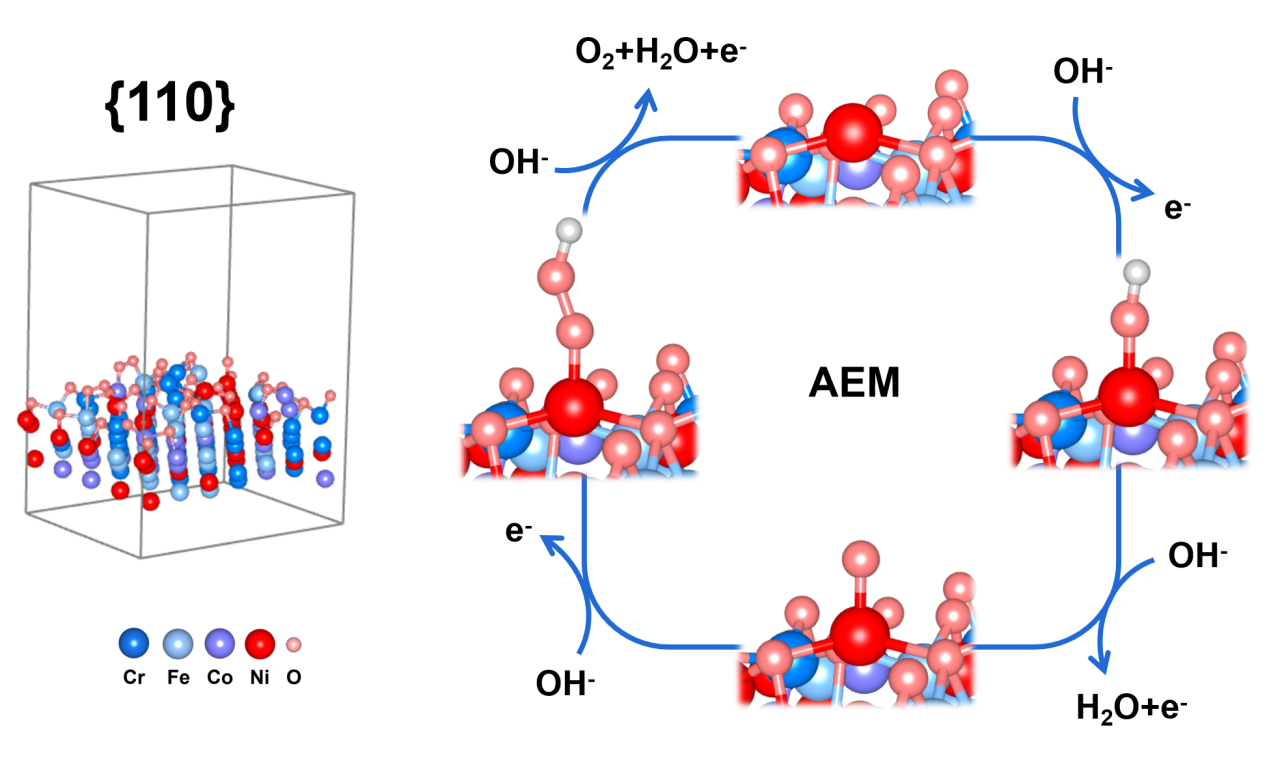


**Figure S22.**Structural modelling and AEM diagrams of {110} facets.


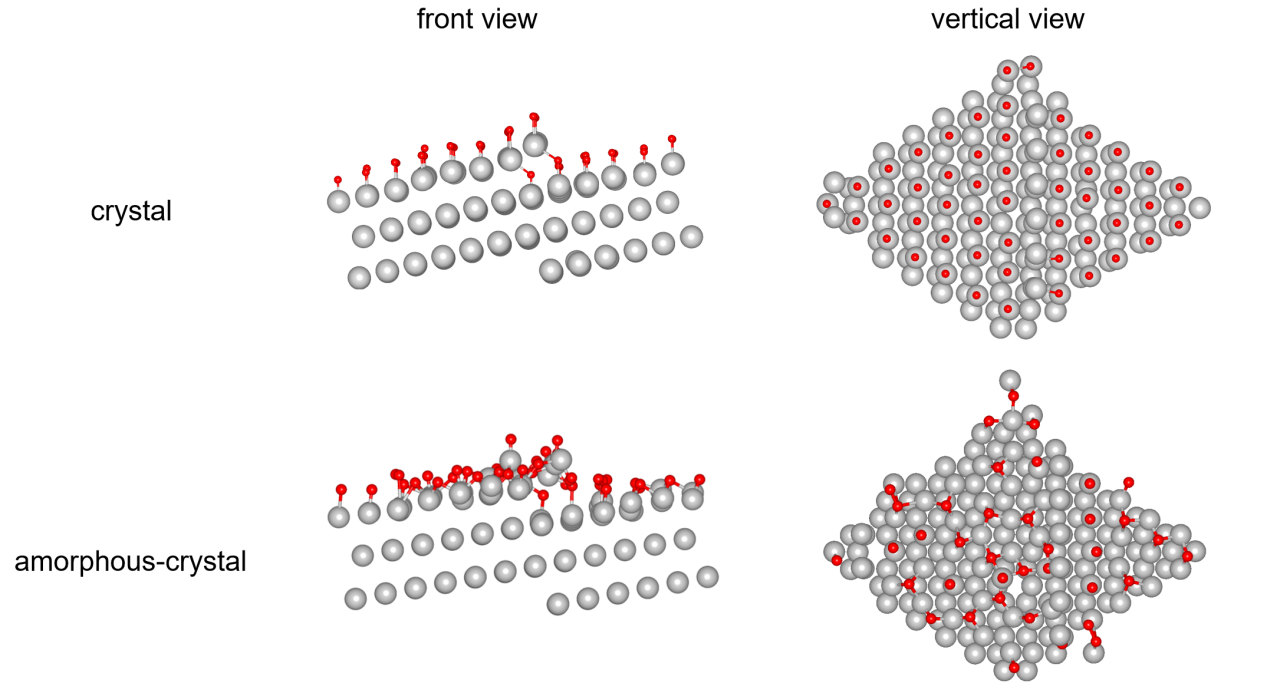


**Figure S23**. Crystal and amorphous-crystal structural modelling of {111} facets.


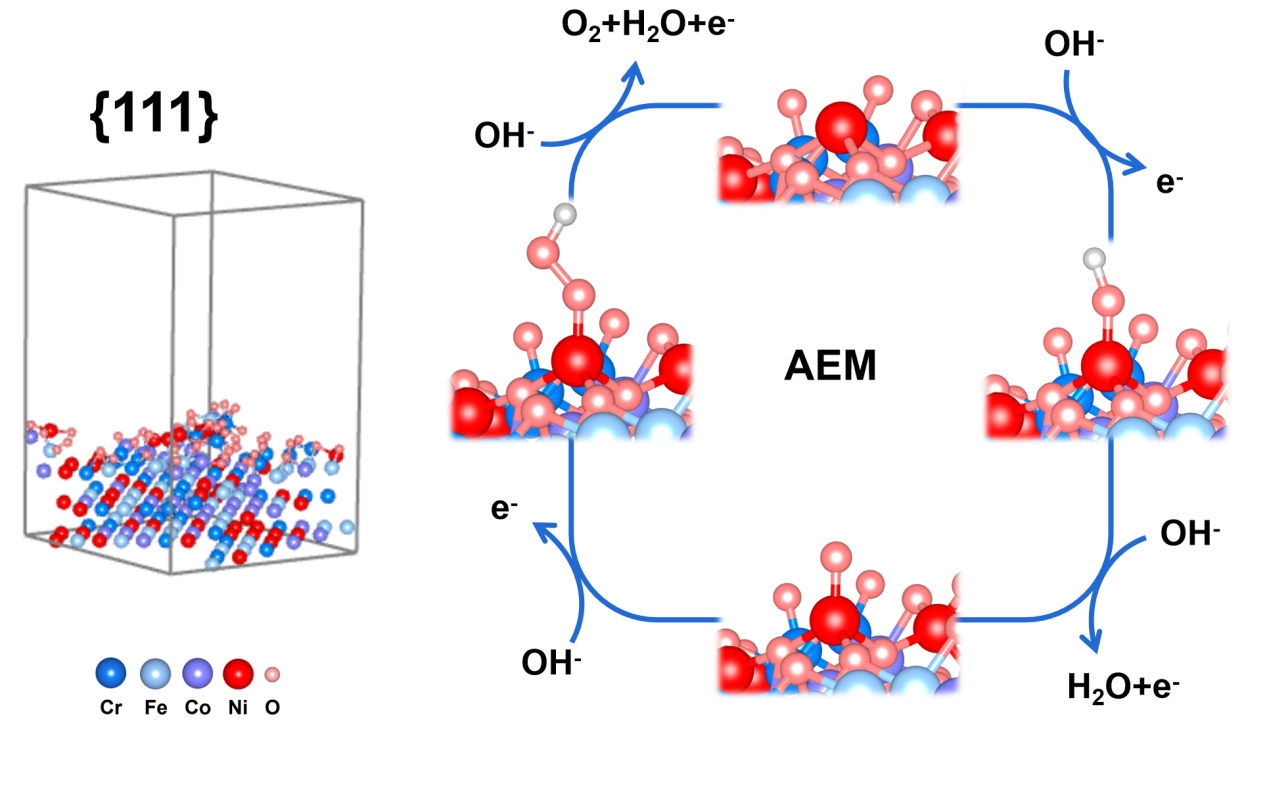


**Figure S24.** Structural modelling and AEM diagrams of {111} facets.


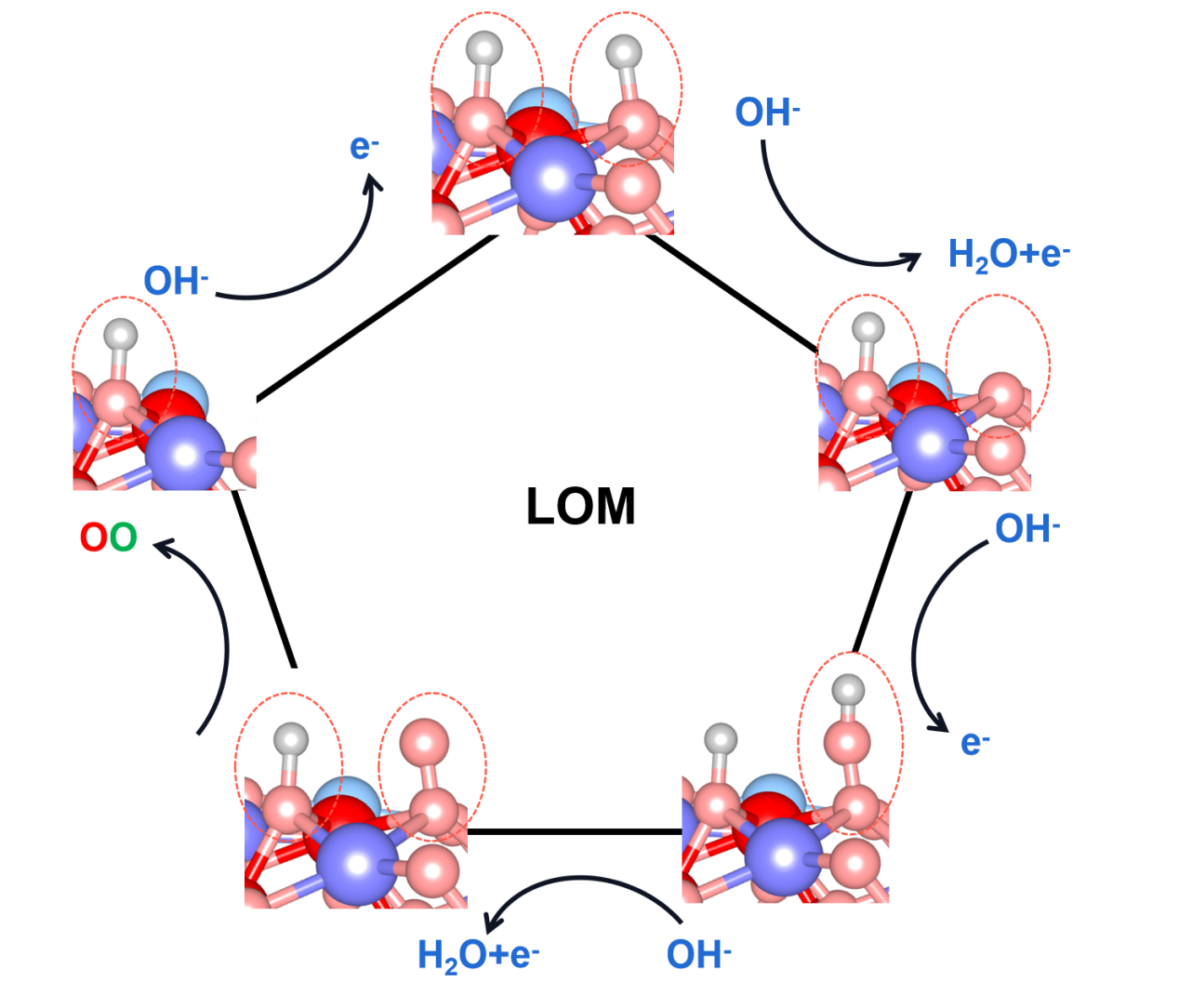


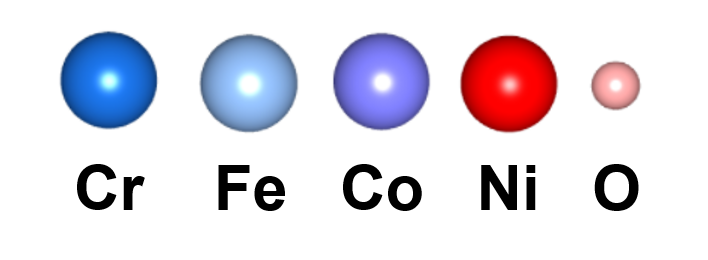


**Figure S25**. LOM diagrams of {100} facets.


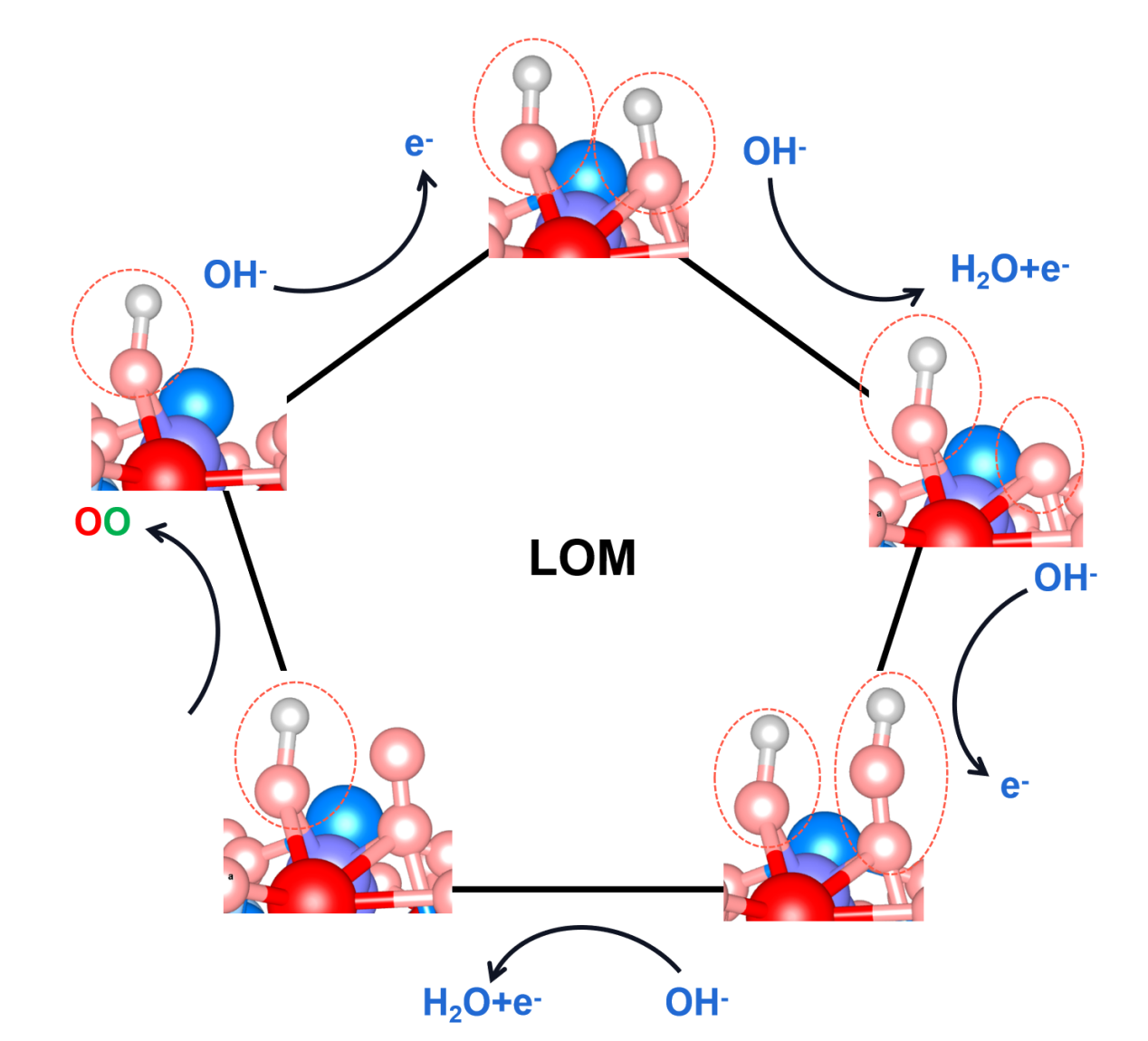


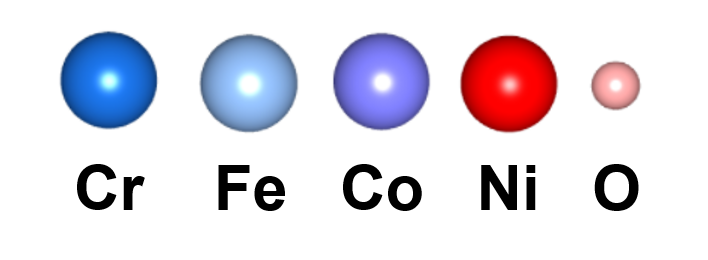


**Figure S26**. LOM diagrams of {110} facets.


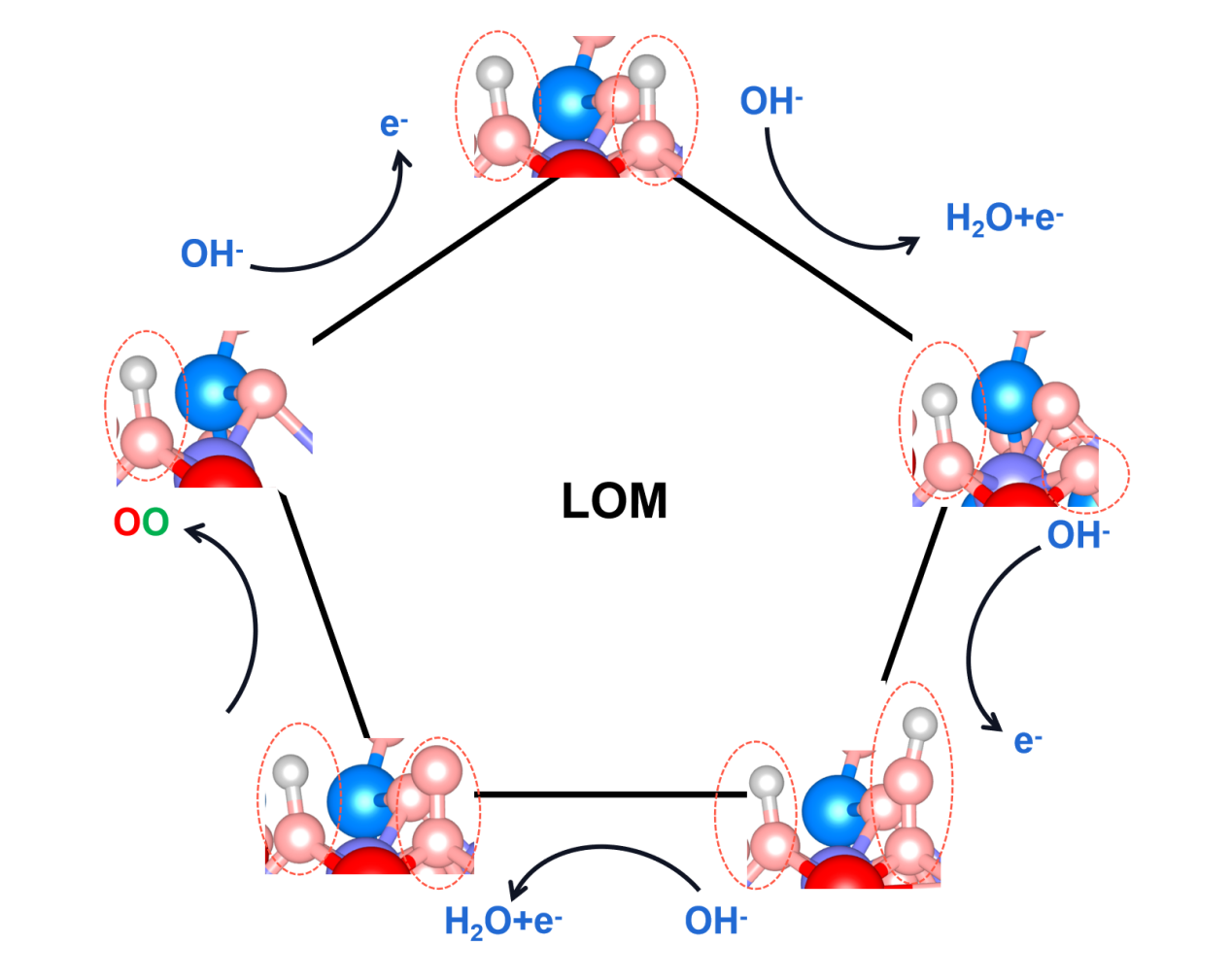


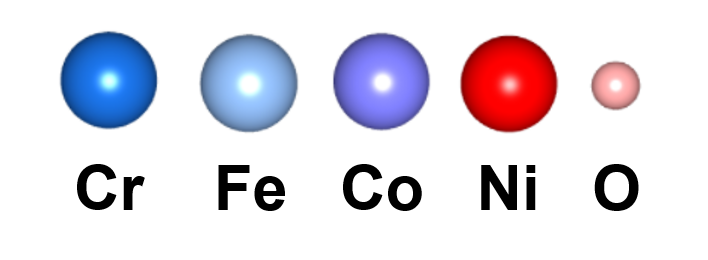


**Figure S27.** LOM diagrams of {111} facets.

[1] H. Wu, T. Yang, Y. Du, L. Shen, G. W. Ho, *Advanced Materials* **2018**, 30, 1804341.

[2] C.-H. Kuo, I. M. Mosa, S. Thanneeru, V. Sharma, L. Zhang, S. Biswas, M. Aindow, S. Pamir Alpay, J. F. Rusling, S. L. Suib, J. He, *Chemical Communications* **2015**, 51, 5951.

[3] L. Liu, Z. Jiang, L. Fang, H. Xu, H. Zhang, X. Gu, Y. Wang, *ACS Applied Materials & Interfaces* **2017**, 9, 27736.

[4] Z. Chen, C. X. Kronawitter, B. E. Koel, *Physical Chemistry Chemical Physics* **2015**, 17, 29387.

[5] L.-L. Feng, G. Yu, Y. Wu, G.-D. Li, H. Li, Y. Sun, T. Asefa, W. Chen, X. Zou, *Journal of the American Chemical Society* **2015**, 137, 14023.

[6] K. Dang, S. Zhang, X. Wang, W. Sun, L. Wang, Y. Tian, S. Zhan, *Nano Research* **2021**, 14, 4848.

[7] X. Han, G. He, Y. He, J. Zhang, X. Zheng, L. Li, C. Zhong, W. Hu, Y. Deng, T. Y. Ma, *Advanced Energy Materials* **2017**, 8, 1702222.

[8] K. A. Stoerzinger, O. Diaz-Morales, M. Kolb, R. R. Rao, R. Frydendal, L. Qiao, X. R. Wang, N. B. Halck, J. Rossmeisl, H. A. Hansen, T. Vegge, I. E. L. Stephens, M. T. M. Koper, Y. Shao-Horn, *ACS Energy Letters* **2017**, 2, 876.

[9] S. Geng, L. Chen, H. Chen, Y. Wang, Z.-B. Ding, D. Cai, S. Song, *Chinese Journal of Catalysis* **2023**, 50, 334.

[10] Y. Tong, Y. Guo, P. Chen, H. Liu, M. Zhang, L. Zhang, W. Yan, W. Chu, C. Wu, Y. Xie, *Chem* **2017**, 3, 812.

[11] S. H. Chang, N. Danilovic, K.-C. Chang, R. Subbaraman, A. P. Paulikas, D. D. Fong, M. J. Highland, P. M. Baldo, V. R. Stamenkovic, J. W. Freeland, J. A. Eastman, N. M. Markovic, *Nature Communications* **2014**, 5, 4191.

[12] A. Fungerlings, M. Wohlgemuth, D. Antipin, E. van der Minne, E. M. Kiens, J. Villalobos, M. Risch, F. Gunkel, R. Pentcheva, C. Baeumer, *Nat Commun* **2023**, 14, 8284.
